# Personality differentially affects individual mate choice decisions in female and male Western mosquitofish (*Gambusia affinis*)

**DOI:** 10.1371/journal.pone.0197197

**Published:** 2018-05-15

**Authors:** Bo-jian Chen, Kai Liu, Lin-jun Zhou, Guilherme Gomes-Silva, Carolin Sommer-Trembo, Martin Plath

**Affiliations:** 1 College of Animal Science and Technology, Northwest A&F University, Yangling, P.R. China; 2 Department of Geography (“Saude Ambiental”), Universidade Federal de Uberlândia, Minas Gerais, Brazil; 3 Department of Ecology and Evolution, J.W. Goethe University Frankfurt, Max-von-Laue-Straße, Frankfurt am Main, Germany; University of Vienna, AUSTRIA

## Abstract

Consistent individual differences in behavioral tendencies (animal personality) can affect individual mate choice decisions. We asked whether personality traits affect male and female mate choice decisions similarly and whether potential personality effects are consistent across different mate choice situations. Using western mosquitofish (*Gambusia affinis*) as our study organism, we characterized focal individuals (males and females) twice for boldness, activity, and sociability/shoaling and found high and significant behavioral repeatability. Additionally, each focal individual was tested in two different dichotomous mate choice tests in which it could choose between computer-animated stimulus fish of the opposite sex that differed in body size and activity levels, respectively. Personality had different effects on female and male mate choice: females that were larger than average showed stronger preferences for large-bodied males with increasing levels of boldness/activity (i.e., towards more proactive personality types). Males that were larger than average and had higher shoaling tendencies showed stronger preferences for actively swimming females. Size-dependent effects of personality on the strength of preferences for distinct phenotypes of potential mating partners may reflect effects of age/experience (especially in females) and social dominance (especially in males). Previous studies found evidence for assortative mate choice based on personality types or hypothesized the existence of behavioral syndromes of individuals’ choosiness across mate choice criteria, possibly including other personality traits. Our present study exemplifies that far more complex patterns of personality-dependent mate choice can emerge in natural systems.

## Introduction

Ever since Darwin proposed sexual selection from female mate choice [1], a plethora of studies have considered the questions of what phenotypic traits are selected by female choice and what mechanisms explain the existence of those preferences [2–4]. For several species, we have a fairly good understanding of why and how choosing individuals (both females and males) perceive and respond to various phenotypic traits of potential mating partners, including morphological traits [5,6], color ornaments [7–9], acoustic signals [10–12] and behavioral displays, i.e., ritualized courtship behaviors [6,13,14]. Recently, there has been increasing interest as to whether and how consistent individual differences in behavioral tendencies—also referred to as animal personality [15]—affect individual mate choice decisions [16–18]. Empirical studies in this direction mainly focused on how personality traits may influence female mate choice decisions, even though behavioral repeatability was not assessed in all cases; they either investigated the effects of male personality traits as a potential mate choice criterion [19–21] or how choosing females’ personality type affects their mating decisions [18,22]. Comparatively few studies considered personality effects during male mate choice [23,24]. Furthermore, there is evidence that personality types of both mating partners can interact to determine mating decisions. This could result in assortative mating, where individuals prefer to mate with partners of a similar personality type [17,25], even though behavioural convergence could also result in similarity of personality types in monogamous and pair-bonding species [26,27].

In our present study we asked whether the choosing individual’s personality affects mate choice in the same way (and to the same extent) in both sexes of the same species, and whether potential personality effects are consistent across different mate choice situations. We used western mosquitofish (*Gambusia affinis*) to answer these questions. Livebearing fishes of the family Poeciliidae show internal fertilization and have a polygamous mating system. They have emerged as model organisms to study sexual selection [28–32]. Both sexes express some degree of mate choice: males of several species try to coerce copulations with certain female phenotypes [33–35]. Females determine the success of those mating attempts by influencing copulation duration (i.e., by seeking or avoiding the proximity of certain male phenotypes [36–38]) and through post-copulatory sperm selection mechanisms [39–41].

We characterized female and male *G*. *affinis* for the following three personality traits: (1) boldness, determined as latency time to emerge from shelter [42–44], (2) general activity [16,18], and (3) sociability, assessed as time spent in the vicinity of a shoal of conspecifics [45,46]. The same focal individuals were subsequently tested in two dichotomous mate choice tests. Stimulus fish of the opposite sex differed in (*a*) body size, and (*b*) locomotor activity, respectively. Numerous studies on poeciliid fishes (including mosquitofish) found both males and females to prefer large-bodied mating partners [47–51], and so we expected to find an overall preference for large mating partners in *G*. *affinis*. Information on potential effects of the choosing individual’s personality on the strength of preference (SOP) for large mating partners is scarce. However, a recent study on another poeciliid species (*Poecilia mexicana*) found highly explorative females to exhibit stronger SOP for large-bodied mating partners than less explorative ones [18]. Exploration is one of the most examined personality traits [15,52,53] that has repeatedly been shown to correlate with boldness in different species, especially fishes [43,54–56]. Hence, we assumed that boldness could have a similar effect on mating preferences for large mating partners in *G*. *affinis* females as exploration has in *P*. *mexicana* females. Specifically, we predicted bolder *G*. *affinis* females to exhibit stronger preferences for large-bodied males. Moreover, even though this has not yet been demonstrated in the context of mate choice, bolder individuals may rely more on information they collect individually (private information) instead of using social information. The opposite is true for shier individuals, which tend to rely more on social information [45,57]. Shy individuals, which are typically also more sociable, may benefit from using social information for mate assessment as they tend to reside more in social aggregations; however, social information was not available in our current experimental set-up, which could impair mate assessment of shy and more sociable individuals. Overall then, we predicted that bolder and less sociable females would exhibit stronger SOP for large male body size (*prediction 1*). For male mate choice, we tentatively predicted similar effects of personality as seen for female mate choice (*prediction 2*).

The second mate choice criterion we considered in our present study was general locomotor activity (swimming speed) of mating partners [58–60]. Locomotor activity is tightly linked to metabolic processes [61,62]. Individual variation in locomotor activity partly reflects an individual’s current body condition, with healthy individuals usually being more active than individuals that are, for example, heavily parasitized [63,64]. Still, there is considerable variation in how parasite infections influence activity patterns in different host-parasite systems [65], and parasitized hosts either increased [66] or decreased their activity levels [67], or activity levels were not affected by parasitization [68]. Despite the uncertain link between activity levels and parasite infection, a study on wild brown trout (*Salmo trutta*) found natural selection to favor individuals with high activity levels [69]. In North American red squirrels (*Tamiasciurus hudsonicus*), activity levels were found to correlate with the growth rate of females’ offspring [70]. Those studies suggest that activity levels might be linked to individual fitness differences, and so activity could be used as a potential mating choice criterion, which should result in an overall preference for active mating partners in both male and female *G*. *affinis*.

Activity is also one of the ‘big five’ most investigated animal personality traits [15,71]. In stream water striders (*Aquarius remigis*), assortative mating based on activity levels was reported [72]. In guppies (*P*. *reticulata*), mating pairs with a similar degree of boldness had increased reproductive success [73], which demonstrates that also non-monogamous species without brood care could benefit from assortative mating based on personality types. The question remains whether assortative mating in poeciliid fishes could also be based on personality traits other than boldness, namely, on activity. Therefore, we tested the prediction that more active individuals of both sexes would show stronger SOP for actively moving mating partners than less active individuals (*prediction 3*).

Finally, we tested whether effects of personality on mating preferences would be consistent across different mate choice situations. One possibility would be that individual differences in choosiness are part of a larger behavioural syndrome (‘choosiness syndrome’), which assumes that some individuals are consistently choosier than others across time or across multiple contexts [74,75]. If this was the case, then individuals that show strong preferences in one mate choice situation (mate choice for large-bodied mates) should also show strong preferences in the second mate choice situation (mate choice for more active mates); however, this hypothesis has not yet been tested empirically [75]. Predictions on the effect of personality traits on individual choosiness also vary [76]. However, the results of the aforementioned study on female mate choice in *P*. *mexicana* [18] along with our own predictions for mate choice in *G*. *affinis* (*predictions 1–3*) suggest that personality traits should differentially affect mating preferences in different mate choice situations. Hence, we predicted not to find a pattern congruent with a choosiness syndrome in *G*. *affinis* (*prediction 4*).

## Methods

### Origin and maintenance of test fish

Western mosquitofish (*G*. *affinis*) are widely distributed from the Mississippi drainage in the USA southward to the Río Tamesi drainage in the Estados Unidos de México [33]. The species was introduced to China for malaria prophylaxis after the 1920^ies^ [77]. Test fish used in this study were wild-caught individuals collected in the species’ invasive distribution range in China (in or near the cities of Baoding (N 115°47.62′, E 38°87.03′), Ankang (N 108°80.88′, E 32°72.63′), Hangzhou (N 120°15.58′, E 30°27.70′), Chaozhou (N 116°62.31′, E 23°65.65′) and Beihai (N 109°11.92′, E 21°48.02′) between March and April, 2016. Today, Chinese regulations dictate preventing the release of this species into natural water bodies (Ministry of Environmental Protection of the People's Republic of China 2016, Index No. 000014672/2016-01463), and capturing mosquitofish in the wild does not require any official permit.

**Fig 1:**
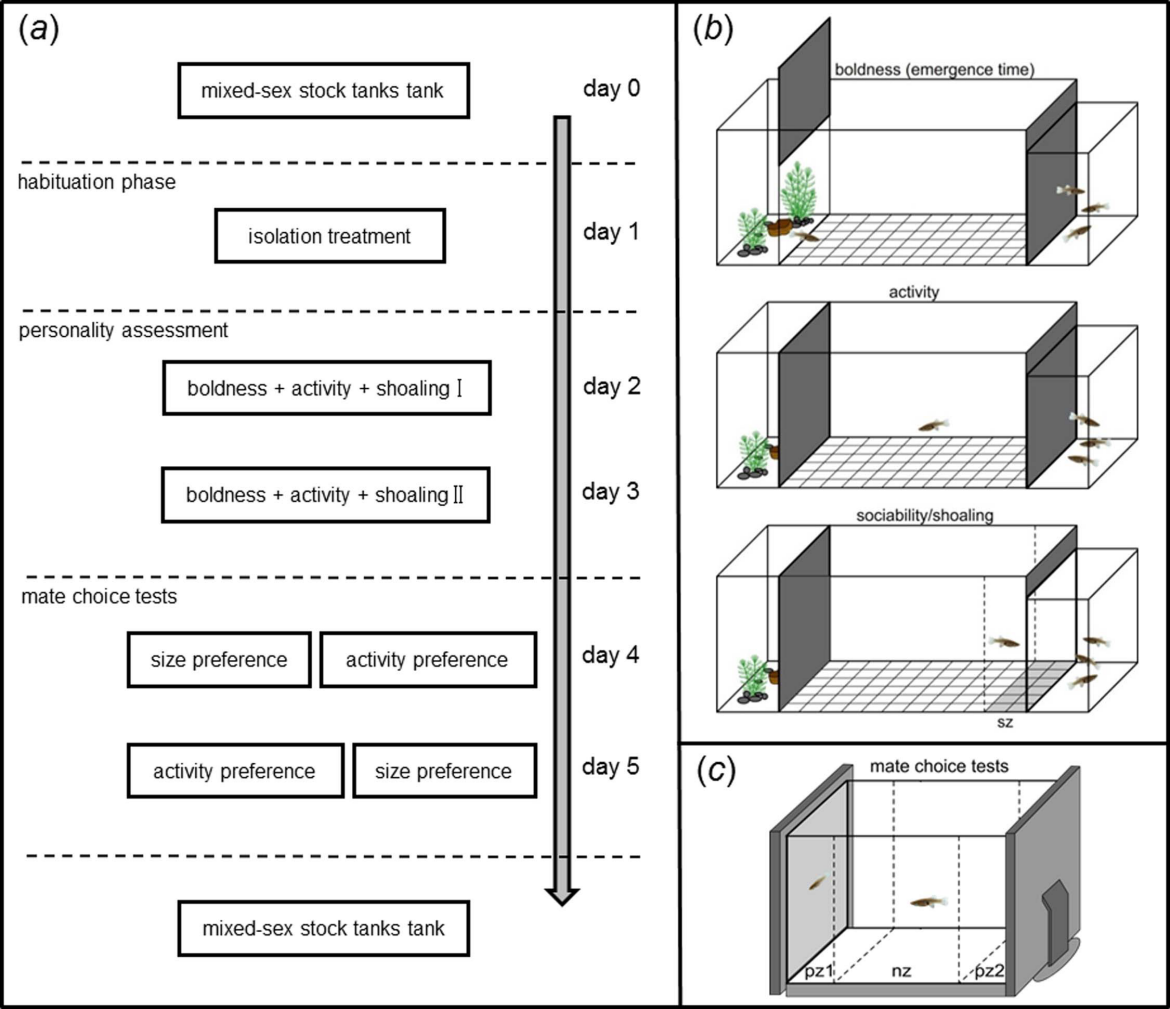
Experimental timeline and schematic view of the experimental set-ups. (*a*) Experimental timeline and (*b*) schematic view of the experimental set-ups used to assess three behavioral measures that are widely used to assess animal personality, namely boldness (assessed as the time focal fish needed to leave the starting zone after the trapdoor was opened), activity (numbers of squares crossed) and sociability/shoaling (time spent in proximity of the stimulus shoal after a divider was removed that had blocked visual contact between the test subject and the stimulus shoal). *sz*, shoaling zone. (*c*) Experimental set-up used to assess focal individuals’ mating preferences as times spent near two monitors showing animated stimulus fish. *pz1*, *pz2*, preference zones; *nz*, neutral zone.

We acclimated the fish to laboratory conditions for at least one month before we conducted behavioral experiments and maintained them in groups comprising both sexes at roughly even sex ratios, at densities of around 40 fish per tank, in several aerated and filtered 200-l aquaria under a 12:12 h light/dark regime. We regularly observed mating behavior in our stock tanks, which often involved females avoiding the coercive mating attempts of certain males, while no pair bonding occurs in *G*. *affinis* [48,50,78]. Even though females’ motivation to mate may decrease when fish are maintained in mixed-sex groups compared with virgins or females that had been isolated from mating partners for some time (e.g., several weeks [79,80], rearing or maintenance conditions do not necessarily affect individuals’ preferences for phenotypic traits of potential mating partners. Thus, studies on mate choice in mosquitofish widely used focal fish originating from mixed-sex tanks [36,81–83]. Aquaria were well equipped with plants, twigs and stones. We fed the fish twice a day *ad libitum* amounts of commercially available flake food, frozen blood worms (chironomid larvae), as well as *Artemia salina* nauplii and shrimps. In the stock tanks and all experimental tanks (see below; Figs 1 and S1), water temperature was kept at 25 ± 1°C. Water quality was maintained by exchanging half of the water every two weeks, while one tenth of the water was exchanged every day in case of the (smaller) isolation tanks (S1 Fig). Aged tap water was used for water changes and throughout the entire experiment.

All experiments reported here comply with current laws and regulations of the PR China and were approved regarding ethics and treatment of animals in research by the Animal Welfare commissioner at the Department of Animal Science of the College of Animal Science and Technology (Commissioner: Dr. Lin-sen Zan; Approval No. 137, 2016).

Behavioral tests were conducted between 22^nd^ May and 30^th^ June 2016. We randomly selected adult focal fish from our stock tanks and isolated them, separated by sex, in 96-l tanks 24 hours prior to behavioral tests. To avoid aggressive interactions between individuals and to enable repeated testing of the same individuals, we kept each focal fish separately in 1.5-l transparent perforated plastic bottles, which allowed water exchange with the environment (see ref. [84] for a study using a similar design; for details see S1 Fig). Furthermore, a longer time in isolation could induce changes in personality in poeciliids [16], and alter males’ motivation to mate [85,86] To avoid such effects, we chose a rather short isolation time of only 24 h. Fish were returned to their isolation tanks between subsequent trials (Fig 1A).

### Behavioral experiments

We firstly characterized each focal fish for three standard indicators of personality using well-established experimental approaches: (1) boldness as latency to emerge from shelter and enter an unknown area [44,87,88], (2) activity in an open field tank [89,90] and (3) sociability (i.e., shoaling tendencies), estimated as the time spent in the vicinity of a group of conspecifics [91,92]. We tested each fish twice for its personality on two successive days, which allowed us to test for behavioral repeatability across both assessments [93]. In the related *Xiphophorus birchmanni*, short time-intervals between repeated personality assessments well reflected long-term behavioral consistency [94]. Afterwards, we used the same test subject and conducted two dichotomous mate choice tests in which each focal individual could choose between two stimulus fish of the opposite sex that differed in (*a*) body size or (*b*) activity levels (for experimental time line see Fig 1A). To avoid carryover effects [95], the order of both preference tests was balanced.

A web cam (KC-QB960AK, Keeper, Shenzhen, China) was fixed in a central position approximately 70 cm above the test tank during all behavioral observations, allowing us to remotely observe the focal fish from above. We introduced an air stone connected to an air pump into the test tanks between trials to guarantee well-oxygenated water, and we changed the water every day after a testing session (every 4 trials). We initially tested *n* = 42 females and *n* = 42 males for their personality. During the subsequent preference tests, one female behaved abnormally, i.e., it continued to show fast-start swimming and other flight responses. Another female was erroneously tested twice for its preference for male body size. Data from those two females were discarded, resulting in a final sample size of *n* = 40 females (mean ± SD, SL: 29.75 ± 4.86 mm) and *n* = 42 males (SL: 23.48 ± 3.02 mm). All focal individuals were measured for standard length (SL) upon completion of a test sequence before they were transferred back into their original stock tanks, after which they played no further role for subsequent tests.

#### Personality assessment

The test arena consisted of a glass tank (80 × 30 × 30 cm) that was filled with aged tap water to a height of 15 cm (Fig 1B). The tank was placed on a gray plastic sheet with a fine white grid (5 cm squares). All outer sides were covered with black plastic foil to minimize disturbance. To initiate a trial, we introduced the focal individual into a lateral shelter area (20 × 30 cm), which was separated from the rest of the tank by an opaque trap door (Fig 1B). The shelter area contained small stones and artificial plants for the fish to hide. We gave the focal fish 2 min for acclimatization before the trapdoor was remotely opened by a pulley system. We determined the latency the test subject needed to emerge from shelter, which is a common measure of boldness in fish, with bolder fish emerging faster [24,42,43]. We terminated a trial after a maximum ceiling value of 5 min (i.e., if the focal fish did not leave the starting area) and gently moved the fish outside the container with the help of a small aquarium dip net (this concerned two males in one of the two repeated measurements each). Afterwards, we closed the trapdoor and let the fish explore the tank for 5 min before we started quantifying swimming activity. This habituation period was important as we were interested in individuals’ activity levels rather than exploration of a novel environment. Even shorter periods of time for habituation were successfully employed in studies in individuals’ activity levels in other poeciliid species [16,96]. We counted numbers of squares crossed by the focal fish in the test arena (60 × 30 cm) within 5 min, assuming that more active fish would cross more grid squares [16,97,98].

Directly after the activity assessment, we gently removed a black cardboard divider that had blocked visual contact with a stimulus shoal that was situated in another tank (20 × 20 × 15 cm), adjacent to the small side of the test tank opposite of the starting area (Fig 1B). Physical and chemical contact between fish residing in different tanks was not possible, leaving only visual cues as a potential stimulus. The tank contained three stimulus fish (SL, females: 28.46 ± 5.41 mm; males: 22.74 ± 3.33 mm), which were exchanged between trials. To avoid effects of sexual attraction and familiarity on shoaling behavior [46,99], we presented stimulus shoals of the same sex, and fish used to compose stimulus shoals were taken from a different stock tank than the one from which the focal fish stemmed. We waited until the focal individual habituated to the new situation and resumed swimming freely. During a 5 min observation period, we determined the time the focal individual spent within a visually marked association zone (10 cm in front of the stimulus tank; Fig 1B) as a measure of sociability/shoaling [91,92]. All tests were performed consecutively in the same arena to minimize handling stress. Our protocol for the assessment of personality variation can be found at https://doi.org/10.17504/protocols.io.m68c9hw.

#### Assessment of mating preferences using computer animations

Computer animation has been successfully applied to study animal behaviour in various contexts and in an array of species (e.g., [100–102]. Even though computer-animated stimuli have not yet been applied to study mate choice in *G*. *affinis*, this technique was successfully used in another context using our study species [103]. Moreover, computer animations were successfully used to assess mating preferences in other poeciliid species [104,105]. For our study, we used computer-animated stimuli for both mate choice tests. Stimulus pairs showed two images of the same individual, which we manipulated in a way that they differed by (*a*) body size and (*b*) locomotor activity, but not in other morphological or behavioural traits that could affect mate choice decisions [101]. Each computer animation showed one virtual stimulus fish swimming in a straight line from left to right and back in front of a uniformly light grey background, with an invisible turn of one body length before changing swimming direction (i.e., we let the animated fish continue swimming outside the display window for one body length and then turn around without being seen by the focal fish [104]).

In order to generate animation pairs that represent natural variation in body size and activity levels (swimming speed) of our study species, we measured the standard length (SL) of *n* = 268 wild-caught *G*. *affinis* (*n* = 141 females and *n* = 127 males) from ethanol-stored samples collected from the abovementioned populations. Body size ranges were established as 20–44 mm (mean ± SD: 29.10 ± 4.67 mm) for females and 15–29 mm (mean ± SD: 22.30 ± 2.86 mm) for males. Moreover, we assessed activity levels of an additional *n* = 72 fish in the laboratory (*n* = 42 females and *n* = 30 males) using the same experimental set-up for activity assessment as described above. By counting the numbers of squares (5 × 5 cm) crossed within 300 s, we estimated swimming speed [cm s^-1^] as: number of squares crossed × 5 / 300. Activity levels were thus established as 0.95–7.2 cm s^-1^ (mean ± SD: 2.71 ± 1.22 cm s^-1^) for females and 1.01–4.53 cm s^-1^ (mean ± SD: 2.63 ± 0.87 cm s^-1^) for males. Data from those pre-trials merely served as a reference to produce the animations and were not included in later analyses, nor were fish used in these assessments retested in the main behavioral experiment.

#### Preparation of animations

To generate animations, we used high resolution photos showing wild-caught, laboratory-maintained individuals in lateral view. We placed individual fish in a small tank (20 × 15 × 15 cm) in front of a light gray background. The tank was filled with aged tap water of 25 ± 1°C to a level of 10 cm height. Photos were taken under natural light conditions while avoiding direct sunlight using a Canon 650D digital camera (Canon, Tokyo, Japan), positioned 30 cm in front of the tank. We took photos after the fish had habituated to the new situation and resumed swimming freely. Altogether, we thus obtained *n* = 48 photos (24 females and 24 males; S2 Fig) and saved them as .jpeg files. From each picture, we extracted the image of the stimulus fish from the background using the “magic extractor” function in Adobe Photoshop CS4. The resulting images were animated and converted into .flv files (resolution 1024 × 768; 30 frames s^-1^) using Macromedia Flash 8. Fish used for generating the animations were not used in the behavioral experiments.

From each of the 48 individual images, four animations were generated (i.e., two animation pairs: large vs. small body size with the same activity level (average swimming speed for a given sex) and high vs. low activity level with the same body size (average SL for a given sex). We defined ‘large’ body size and ‘high’ activity as the empirical mean values (for a given sex) plus the respective standard deviation (see above), while ‘small’ body size and ‘low’ activity were defined as the empirical mean values minus the associated SD-values.

#### General testing procedure

The experimental set-up for the dichotomous association preference tests consisted of a tank (60 × 30 × 35 cm) with two computer screens (L1510A, Lenovo, Beijing, China) placed on both smaller sides (Fig 1C). We set the two screens to the same calibration configuration to achieve uniform display properties with respect to brightness and hue. The test tank was visually divided into three sections: two preference zones (10 cm) adjacent to the screens and a central neutral zone (40 cm). Both long sides of the tank were covered by black plastic foil to minimize outside disturbance. We filled the tank with water to a level of 25 cm, which matched the height of the screens. Illumination was provided by a 35 W LED lamp 40 cm above the tank in addition to diffuse room illumination.

To initiate a trial, we introduced the focal individual into a clear Plexiglas cylinder (10 cm diameter), placed centrally into the neutral zone of the tank, and started playback of the first pair of animations. After a 5 min habituation period, during which the fish could see both animations, we gently removed the cylinder. During the following 5 min observation period we measured association times, i.e., times spent in each preference zone [104,106,107]. Association time in this experimental situation has been demonstrated to be a good indicator of female mating preferences in related species [5,51,108–110]. To avoid potential side-biases, we retransferred the focal fish into the central cylinder, interchanged side-assignments of the stimulus animations, and repeated measurement of association preferences after another 5 min for habituation. The second mate choice test was conducted on the next day (for testing order see Fig 1A). Our protocol for the assessment of mate preferences based on computer animation can be found at https://doi.org/10.17504/protocols.io.m67c9hn.

### Statistical analyses

All statistical analyses were conducted in SPSS 19. All descriptive statistics are presented as mean ± SE values. Raw data of the study can be found in the Online Supplementary Material (S1 file)

#### Repeatability of personality traits and behavioral syndromes

A common way to quantify the degree of consistency of repeatedly measured traits is to calculate repeatability (*R*)-values, defined as: *R* = variance among individuals / (variance among individuals + variance within individuals) [93]. Data from our activity assessment showed a normally distributed (Gaussian) error structure and so variance estimates could be obtained from a linear mixed model (LMM). We used a generalized linear mixed model (GLMM) to obtain variance estimates of the data from our assessment of boldness, which showed a *γ*-shaped error structure. Significant deviations of *R* from zero were tested using Wald’s *z*-tests. Data from our assessment of shoaling tendencies did not meet any distribution pattern that would allow inclusion as dependent variable in a LMM or GLMM, and no transformation could improve the distribution. As an alternative estimate of behavioral consistency, we compared data from the two assessments using non-parametric Spearman rank correlations.

To test for correlations between personality traits, we used phenotypic correlations—an approach that has recently been shown to adequately capture behavioral syndrome structures [111]. To this end, we used mean values from both assessments and calculated Pearson correlations (after checking that the error structure was normally distributed). We corrected *α-*levels for multiple testing as α*’* = 0.05 / 2 = 0.025. For further analyses, we condensed single personality traits using a principal component analysis (PCA): a correlation-matrix based PCA retrieved a single PC with an eigenvalue > 1 from the data on female personality and two PCs from the data on male personality traits (for details see Table 1). Finally, we tested for a correlation of SOP-values from both mate choice situations (i.e., “choosiness syndrome”) by means of Pearson correlations for each sex, separately.

**Table 1:** Results of correlation matrix-based principal component analyses using three different personality traits as input variables.

	Females	Males	
Principal Component (PC)	PC1	PC1	PC2
Eigenvalue	1.56	1.47	1.03
%Variance explained	52.10	48.87	34.37
Boldness (emergence time)	–0.79	–0.76	–0.42
Activity	+0.84	+0.89	-0.17
Shoaling	+0.49	+0.01	+0.96

PCAs were run for females and males, separately. Shown are axis loadings for PCs with eigenvalues > 1.

#### Strength of mating preferences and influence of personality

We estimated each focal individual’s strength of preference (SOP) for large versus small and active versus less active mating partners as:

SOP = [time spent associating with large (or active) stimulus fish–time near small (or less active) stimulus fish)] / time spent with both stimulus fish.

Thus, SOP-values could range from +1 (preference for large-bodied or active individuals) to -1 (preference for small-bodied or less active individuals). To test for overall preferences within each group and test situation, we compared SOP-values against a null assumption (i.e., SOP = 0) by using one-sample *t*-tests. Afterwards, we used general linear models (GLM) to estimate the effects of our measures of animal personality on among-individual variation in mating preferences. SOP-values were used as the dependent variable, while personality-related PCs of the focal individual and focal individuals’ SL were included as covariates. We decided to include personality-related PCs and SL as separate covariates as we detected no significant correlations between body size and any of our measures of personality (Pearson correlations, females: *r*_P_ < 0.093, *P* > 0.55, *n =* 41; males: *r*_P_ < 0.11, *P* > 0.51, *n* = 42). Two-way interactions were initially included, but were excluded from the final models if non-significant (female preference for active males: PC1 × SL: *F*_1,36_ = 1.52, *P* = 0.23). Inclusion of SL as a covariate and inclusion of two-way interaction terms was motivated by the finding that focal females’ SL significantly affected their SOP for large mating partners in the related *P*. *mexicana* (i.e., SOP for large males decreased with increasing SL [18]. We, therefore, hypothesized that personality effects might manifest differently in focal individuals of different body size classes, leading to significant interaction effects. We initially coded ‘animation ID’ as a random effect. However, given that overall effect sizes were relatively low in our models, we decided to prioritize main and interaction effects based on previous studies. To avoid over-parameterization of model structures, we excluded non-significant interaction terms (see above) as well as the random effect (*P* > 0.16 in all cases) from the final models.

## Results

### Repeatability of personality traits and behavioral syndrome structures

We found high and significant estimates of repeatability (*R*) for boldness (i.e., emergence times; females: *R* = 0.56, 95% CI [0.51; 0.61]; Wald’s *z* = 3.14, *P* = 0.002; males: *R* = 0.59, 95% CI [0.54; 0.63], *z* = 3.24, *P* = 0.001) and activity (numbers of squares crossed; females: *R* = 0.78, 95% CI [0.77; 0.79]; *z* = 3.94, *P* < 0.001; males: *R* = 0.53, 95% CI [0.48; 0.59]; *z* = 3.01, *P* = 0.003). For sociability, we estimated behavioral consistency via Spearman rank correlations. For males, we found sociability-scores to be significantly correlated between both assessments (*r*_S_ = 0.72, *P* < 0.001, *n* = 42), suggesting behavioral consistency, while a non-significant trend was detected in the case of female focal individuals (*r*_S_ = 0.26, *P* = 0.099, *n* = 42).

Descriptive statistics, as well as statistical comparisons of mean values between sexes, can be found in the Online Supplementary Material (S1 Table).

**Fig 2:**
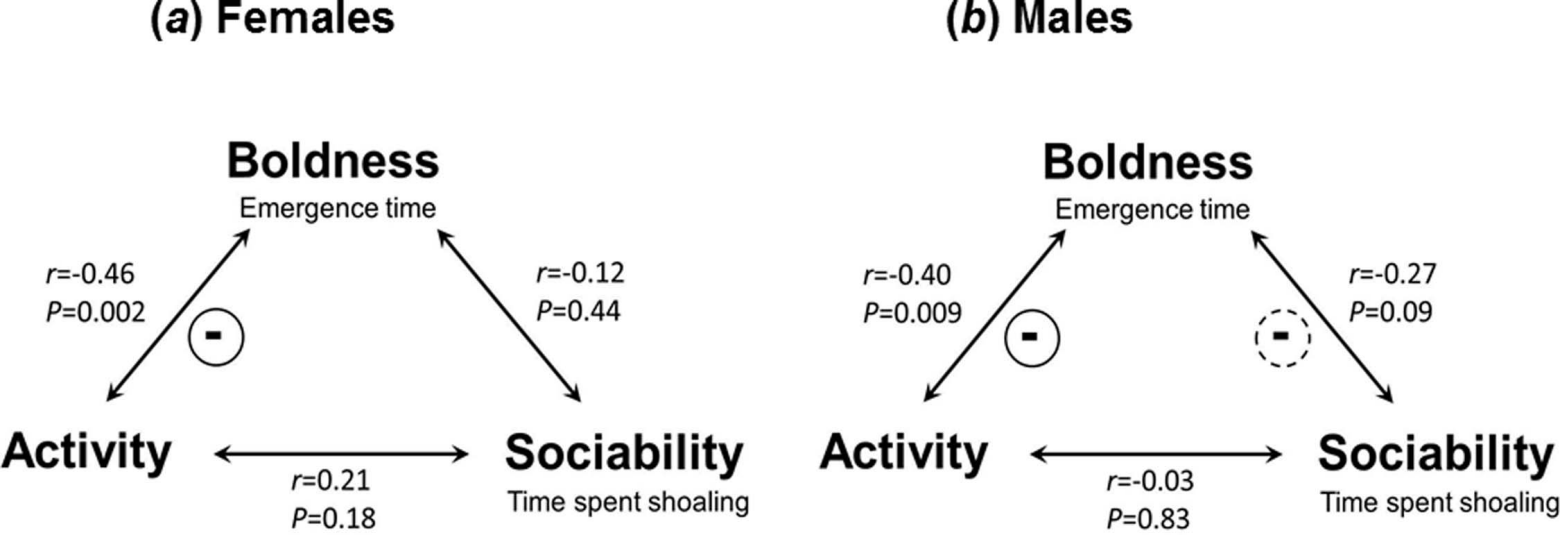
Behavioral syndrome structures of focal (*a*) females and (*b*) males, assessed via Pearson correlations between the three behavioral parameters (boldness, activity and sociability/shoaling tendencies).

When we tested for behavioral syndrome structures, we found negative correlations between emergence times and activity in both sexes (Pearson correlations, females: *r*_p_ = -0.46, 95% confidence interval, CI [-1.27; -0.31], *P* = 0.002, *n* = 42; males: *r*_p_ = -0.40, 95% CI [-0.46; -0.071], *P* = 0.009, *n* = 42; Fig 2A and 2B). It has been reported that bold individuals of our study species show short emergence times, while shy individuals exhibit longer emergence times [50], and so our results suggest that bolder individuals were also more active. No significant correlations were found between other personality traits (Fig 2A and 2B), even though there was a suggestive trend for a negative correlation between emergence times and our measure of sociability in males (*r*_p_ = -0.27, 95% CI [-0.73; 0.05], *P* = 0.09, *n* = 42). Results of PCA confirmed this pattern (Table 1): one PC was retrieved from female personality data with high axis loadings from boldness (-0.79) and activity (+0.84), but only a weak loading from shoaling (+0.49). Two PCs were retrieved from male personality data. PC1 received high axis loadings from boldness (-0.76) and activity (+0.71) and only a weak loading from shoaling (+0.45), while PC2 received weak loadings from boldness (-0.03) and activity (+0.57), but a high loading from shoaling (+0.84).

### Overall direction of mating preferences

**Fig 3:**
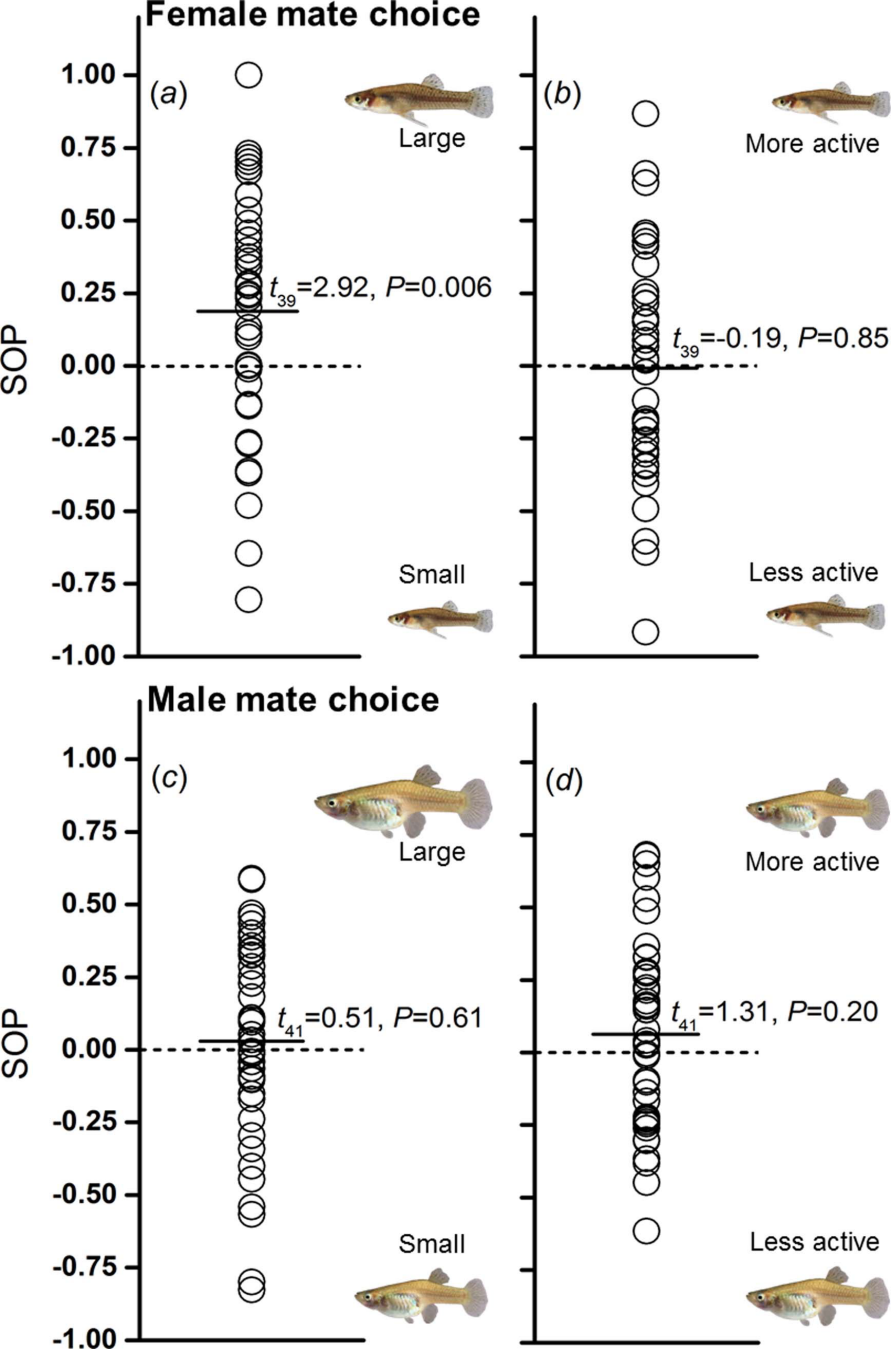
Distribution of individual strength of preference (SOP)-values derived from dichotomous association preference tests. (*a*) female choice for large versus small male body size, (*b*) female choice for active versus less active males, (*c*) male mate choice for large versus small female body size, and (*d*) male choice for active versus less active females. Solid lines represent the mean SOP across individuals. Results from one-sample *t*-test testing against the null assumption (SOP = 0) are presented.

We initially expected both sexes to show preferences for large-bodied mating partners. However, a statistically significant effect was detected only in females, i.e. females spent, on average, significantly more time in association with the animations showing large-bodied males (189.73 ± 12.90 s) than with small-bodied males (138.18 ± 13.06 s; *t*_39_ = 2.92, 95% CI of the difference [0.058; 0.32], *P* = 0.006; Fig 3A). Males did not show an overall preference for large (173.67 ± 11.21 s) over small-bodied females (157.26 ± 9.67 s; *t*_41_ = 0.51, 95% CI [-0.082; 0.14], *P* = 0.61; Fig 3C).

We also expected both sexes to show overall preferences for active mating partners. Pronounced variation in individual preferences (i.e., SOP-values) was observed; however, no significant overall preferences were detected. Females did not spend more time near males showing high activity levels (164.55 ± 12.34 s) compared to males with low activity levels (168.35 ± 12.70 s; *t*_39_ = -0.19, 95% CI of the difference [-0.14; 0.11], *P* = 0.85; Fig 3B). Likewise, males spent similar amounts of time near females showing high activity levels (186.10 ± 12.03 s) and females showing low activity levels (160.45 ± 9.81 s; *t*_41_ = 1.31, 95% CI [-0.036; 0.17], *P* = 0.20; Fig 3D).

The Online Supplementary Material (S2 Table) shows statistical comparisons of SOP-values for large vs. small, and active vs. less active stimulus individuals between cohorts of focal individuals that we categorized as larger or smaller, and more active or less active than the empirical mean value. Non-significant results were retrieved in all comparisons, suggesting that absence of overall preferences in three out of four cases (see above) was not simply obscured by assortative mating patterns.

### Correlation between personality traits and individual mating preferences

Absence of an overall preference in three out of four test situations (see above) does not preclude the possibility that the observed variation in SOP-values is dependent on personality traits (*predictions 1–3*). We found statistically significant effects of personality-related PCs in two cases: females’ SOP for large male body size (Table 2A) and males’ SOP for more actively swimming females (Table 2D). No statistically significant effects were found in case of females’ SOP for active males and males’ SOP for large female body size (Table 2B and 2C).

**Table 2:** Results of General Linear Models (GLMs) testing for the effects of the choosing individual’s personality type (PCs; Table 2) and body size (SL) of the choosing individual on the strength of female (*a*, *b*) and male preferences (*c*, *d*).

Factor	*B*	95% confidence interval		*df*	*F*	*P*	Wilks’ partial *η*_p_^2^
(*a*) Female preference for large male body size							
**PC1 (boldness, activity)**	**-1.27**	**-2.425**	**-0.116**	**1**	**4.99**	**0.032**	**0.122**
Female body size (SL)	-0.014	-0.041	0.012	1	1.16	0.29	0.031
**PC1 × female SL**	**0.044**	**0.006**	**0.083**	**1**	**5.40**	**0.026**	**0.131**
Error				36			
(*b*) Female preference for active males							
PC1 (boldness, activity)	-0.038	-0.164	0.087	1	0.38	0.54	0.010
Female body size (SL)	-0.005	-0.031	0.022	1	0.14	0.71	0.004
Error				37			
(*c*) Male preference for large female body size							
PC1 (boldness, activity)	-0.70	-1.748	0.348	1	1.84	0.18	0.050
PC2 (shoaling)	1.16	-0.234	2.545	1	2.85	0.10	0.075
Male body size (SL)	-0.028	-0.066	0.011	1	2.12	0.15	0.057
PC1 × PC2	-0.011	-0.104	0.083	1	0.06	0.82	0.002
PC1 × male SL	0.031	-0.013	0.075	1	2.02	0.16	0.055
PC2 × male SL	-0.052	-0.111	0.008	1	3.09	0.088	0.081
Error				35			
(*d*) Male preference for active females							
PC1 (boldness, activity)	-0.22	-1.161	0.719	1	0.23	0.64	0.006
PC2 (shoaling)	-1.19	-2.437	0.056	1	3.76	0.061	0.097
Male body size (SL)	0.024	-0.011	0.058	1	1.92	0.18	0.052
PC1 × PC2	0.043	-0.041	0.127	1	1.08	0.31	0.030
PC1 × male SL	0.009	-0.030	0.048	1	0.21	0.65	0.006
**PC2 × male SL**	**0.055**	**0.001**	**0.108**	**1**	**4.31**	**0.045**	**0.110**
Error				35			

We used SOP-values (see main text) as dependent variables, which were calculated from mate choice tests for large vs. small (*a*, *c*) or active vs. less active stimulus individuals (*b*, *d*). Interaction terms were excluded from the final model if *P* > 0.1 starting with the highest-level interaction term, but the next hierarchical level of interactions was retained if one term had *P* < 0.1 (Note that further exclusion of single interaction terms did not alter the results qualitatively). Significant effects are highlighted in bold typeface.

**Fig 4:**
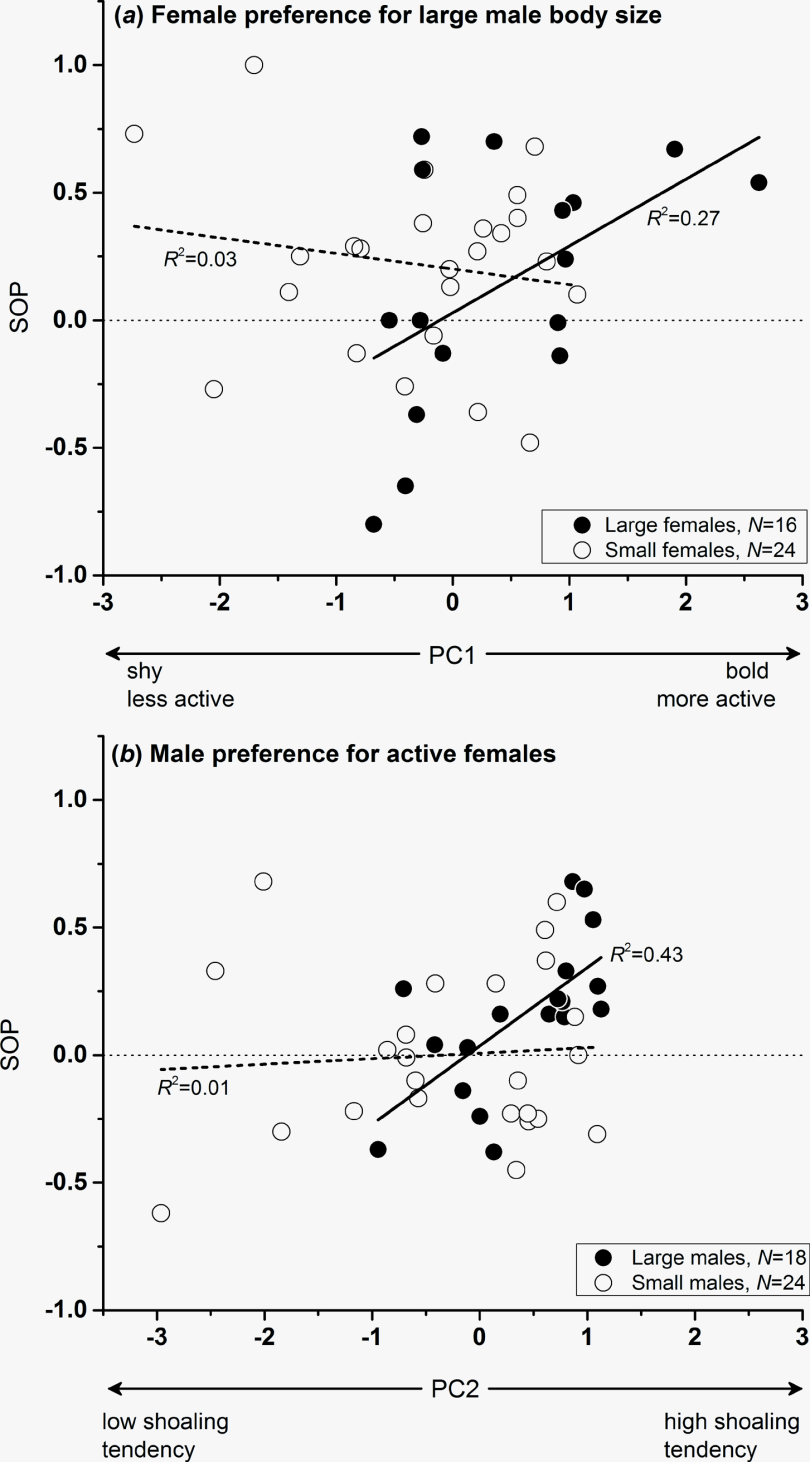
Visualization of significant interaction effects from the GLMs using SOP from (*a*) tests for female choice for large male body size and (*b*) male choice for more actively moving females as the dependent variable. (*a*) Interaction effect between personality traits (PC1; see Table 1) and female body size; (*b*) interaction effect between personality traits (PC2) and male body size. For visualization, data sets are split by the mean empirical body size (SL) of the focal fish (*a*: 29.75 mm; *b*: 23.48 mm).

Females’ SOP for large male body size was influenced by both, the main effect of female personality trait-related PC1, and the interaction effect between female body size (SL) and PC1 (Table 2A). To illustrate the interaction term, we divided the data into two cohorts, in which the body size of focal females was either larger or smaller than the empirical mean value of 29.75 mm. Pearson correlations found the SOP to increase with increasing PC1 (i.e., towards bolder and more active individuals) in females that were larger than average (*r*_P_ = +0.52, 95% CI [0.013; 0.51], *P* = 0.04, *n* = 16), while no such effect was seen in females smaller than the empirical mean (*r*_P_ = -0.17, 95% CI [-0.22; 0.098], *P* = 0.43, *n* = 24; Fig 4A).

In the analysis of male SOP for more active females, we detected a significant interaction effect between male SL and PC2 (Table 2D). There was a suggestive (0.05 < *P* < 0.1) albeit non-significant main effect of PC2 (Table 2D). To visualize the interaction effect, we divided focal males into a large and a small cohort based on the empirical mean SL of 23.48 mm. Pearson correlations revealed that large-bodied males showed an effect of increasing SOP with increasing shoaling tendencies, i.e., increasing values of PC2 (*r*_P_ = +0.66, 95% CI [0.12; 0.49], *P* = 0.003, *n* = 18), while no such effect was seen in smaller males (*r*_P_ = +0.072, 95% CI [-0.11; 0.15], *P* = 0.74, *n* = 24; Fig 4B).

### Absence of a “choosiness syndrome”

When we tested for a behavioral syndrome structure of SOP-values across mate choice situations (i.e., “choosiness syndrome”), we found no significant correlations in both females (*r*_p_ = +0.24, 95% CI [-0.082; 0.59], *P* = 0.13, *n* = 40) and males (*r*_p_ = -0.22, 95% CI [-0.57; 0.10], *P* = 0.17, *n* = 42).

## Discussion

We tested female and male Western mosquitofish (*G*. *affinis*) for their mating preferences regarding two mate choice criteria (body size and locomotor activity), and asked whether the strength of preference (SOP) for different phenotypes is dependent on the choosing individuals’ personality. Our study tested two major questions: do personality traits affect individual variation in SOP similarly in both sexes? Are such personality-effects consistent across different mate choice situations? A sexual motivation of association behaviour became evident at least in the case of male focal individuals, which regularly responded to the animations by showing pre-mating behaviors like attempted gonopodial thrusts, similar to the response to live stimulus females [50]. We found a significant overall preference for large body size only in females, confirming previous studies [33,48,112–114], while males showed no overall preference for large-bodied females (see also [48,115,116]). Neither females nor males showed an overall preference for mating partners with high locomotor (swimming) activity. Still, among-individual variance of SOP-values was high in all cases, leaving the possibility that individuals with a certain personality combination would show positive SOP and others negative SOP, effectively resulting in a net SOP of about zero (a pattern that could emerge if assortative mating played a role [17,25]).

We found different effects of personality on female and male mate choice, which is counter to our original predictions (*prediction 2* and *3*). Females that were larger than average showed stronger SOP for large-bodied males with increasing levels of boldness/activity (in partial fulfillment of *prediction 1*). Males that were larger than average and had higher shoaling tendencies showed stronger SOP for actively swimming females (negating *prediction 3*, which assumed an effect of activity in both sexes). Congruent with *prediction 4*, we found no correlation between SOP-values across mate choice situations (i.e., no evidence of a “choosiness syndrome” involving choosiness across mate choice criteria and possibly other personality traits [74,75]). The latter finding was not surprising as personality affected mate choice differently depending on sex and context (i.e., the experimental mate choice criterion), and personality effects were only observed in large-bodied individuals.

### Personality affects female choice for large male body size

Female preferences for large male body size are widespread in poeciliid fishes [33,48,112–114]. The strong overall preference observed in this study underscores the validity of using animated (virtual) stimuli to study mate choice in fishes [101,104]. By choosing large-bodied males, females not only gain indirect benefits [2,17] but also direct benefits: small-bodied males show more sexual harassment than large-bodied males in *Gambusia* spp. and other poeciliids [112,117,118], a behavior that imposes considerable costs on females [119,120]. Additionally, large males tend to protect females from the sexual harassment of small males [112,118].

Only females that were larger than average showed personality-dependent mate choice, whereby females’ SOP for large-bodied males increased with increasing boldness and activity levels (i.e., towards more proactive personality types). Some studies on poeciliid fishes reported effects of female body size on individual mate choice decisions [113,121,122]. Poeciliid females continue to grow after maturation [48,49], and so female body size should be a good proxy of age. Females could refine their preference for larger males with age via learning either through personal experience or through mechanisms of social learning, e.g., eavesdropping on male contests or mate choice copying [123–127]. Moreover, the “feedback loop theory” [128] assumes personality types to be strengthened over an individual’s lifetime, which implies that personality should have more clear-cut effects on mate choice decisions in older individuals.

But why did bolder and more active females within the cohort of larger-than-average females show stronger preferences? Bolder individuals tend to rely more on private than social information sampling [45,57,129] (but see [130], for context-dependent uncoupling of the link between individual boldness and the extent of social information use). Our experimental design did not allow for social information use during mate choice, and so bolder and more active individuals could have been better and faster at evaluating different mating partners in this particular situation [18]. Following this line of argumentation, we would have expected an effect of boldness/activity on the SOP for male locomotor activity as well. Future studies will need to elaborate on the question of why only males (see below) but not females consider locomotor activity as a mate choice criterion in our study species.

### Personality affects male choice for female locomotor activity

Personality-based assortative mating was most thoroughly studied in monogamous species with biparental brood care, motivated by the hypothesis that preferences for behaviorally similar partners could promote cooperation between mates, thus increasing reproductive success [17,25,131–133]. Subsequent studies found that similarity of behavioral types can also be achieved through behavioral convergence over time [26,27]. Personality-based assortative mating was also reported for non-monogamous species [72,73], which roots our *prediction 3*. However, we did not find evidence for assortative mating based on activity levels: even though *G*. *affinis* males that were larger than average showed personality-dependent mate choice, males’ SOP for females with high locomotor activity increased with increasing sociability (i.e., shoaling tendencies), not activity. Why was a personality-effect observed only in large-bodied focal males? Like in many poeciliids [134,135], somatic growth almost ceases in *G*. *affinis* males upon reaching sexual maturity [48]. Hence, the abovementioned explanations regarding age- and experiential effects fail to explain the observed pattern. Theoretically, larger individuals could also represent more fast-growing genotypes [136]. We argue that large-bodied males usually become dominant in poeciliid social hierarchies [112,137,138], and females prefer large-bodied males [48,112,113]. This leaves large-bodied males with more opportunities to exert mate choice, while smaller males typically adopt sneaky mating tactics [78,117,139] and may be more likely to approach females indiscriminately.

How can the observed personality-effect within the cohort of larger-than-average males be explained? A previous study [50] used a similar experimental approach to quantify sociability in *G*. *affinis* and argued that shoaling tendencies may not necessarily capture sociability in males. Focal males’ decision to approach the stimulus shoal (consisting of three unfamiliar males) was coupled with the decision to accept increased levels of competition, which bolder and more aggressive individuals may be more likely to do. Our result of a suggestive (albeit not statistically significant) correlation between boldness and shoaling tendencies (significant in [50]) lends support to this explanation. Therefore, we tentatively argue that males that are more willing to approach a group of competitors are also more likely to accept increased male competition for more active (i.e., high quality) females [5,39,83].

## Conclusions

Personality affected female and male mate choice in different mate choice situations. This partly reflects that the importance of certain mate choice criteria can differ between both sexes [140]. Moreover, mate competition plays a more important role during male than during female mate choice in poeciliids, as no pair-bonding occurs and males provide no brood care [112]. Along with age- and experiential effects, this brought about a nuanced pattern of personality-dependent mate choice, whereby personality effects were only seen in large-bodied individuals. Finally, several studies provide evidence for assortative mate choice based on personality types [17,23,25,73], or hypothesized the existence of “choosiness syndromes” (correlations of choosiness across mate choice criteria and possibly other personality traits [74,75]). Our present study exemplifies that far more complex patterns of personality-dependent mate choice can emerge in natural systems.

## Supporting information

S1 Fig Schematic view of the isolation tank in which test subjects were maintained before and between the different behavioral assessments. (TIF)

S2 Fig Images of females (F1 –F24) and males (M1 –M24) used to generate computer animations. (TIF)

S1 File Raw data of the study. (XLSX)

S1 Table Results from independent-samples *t*-tests comparing our three measures of personality between female and male *G*. *affinis*. (DOCX)

S2 Table Results from independent-samples *t*-tests comparing SOP-values for large mating partners between large and small individuals and SOP-values for active mating partners between active and less active individuals in (*a*) female and (*b*) male *G*. *affinis*. (DOCX)

## References

[1] Clutton-Brock T. Sexual selection in females. Anim Behav. 2009; 77: 3–11. doi: 10.1126/science.1133311 18096798

[2] Andersson M, Simmons LW. Sexual selection and mate choice. Trends Ecol Evol. 2006; 21: 296–302. doi: 10.1016/j.tree.2006.03.015 16769428 10.1016/j.tree.2006.03.015

[3] Hughes AL. Sexual Selection and Mate Choice: Insights from Neutralist Perspectives. Evol Biol. 2015; 42: 366–378. doi: 10.1007/s11692-015-9315-x

[4] Majerus MEN. The genetics and evolution of female choice. Trends Ecol Evol. 1986; 1: 1–7. doi: 10.1016/0169-5347(86)90056-X 21227769 10.1016/0169-5347(86)90056-X

[5] Herdman EJE, Kelly CD, Godin JGJ. Male mate choice in the guppy *Poecilia reticulata*): Do Males Prefer Larger Females as Mates? Ethology. 2004; 110: 97–111. doi: 10.1111/j.1439-0310.2003.00960.x

[6] Cornuau JH, Rat M, Schmeller DS, Loyau A. Multiple signals in the palmate newt: ornaments help when courting. Behav Ecol Sociobiol. 2012; 66: 1045–1055. doi: 10.1007/s00265-012-1355-y

[7] Amundsen T, Forsgren E. Male mate choice selects for female coloration in a fish. P Natl Acad Sci USA. 2001; 98: 13155–13160. doi: 10.1073/pnas.211439298 11606720 10.1073/pnas.211439298 PMC60840

[8] Gomez D, Richardson C, Lengagne T, Plenet S, Joly P, Lena JP et al The role of nocturnal vision in mate choice: females prefer conspicuous males in the european tree frog (*Hyla arborea*). Pro R Soc B-Biol Sci. 2009; 276: 2351–2358. doi: 10.1098/rspb.2009.0168 19324736 10.1098/rspb.2009.0168 PMC2690462

[9] Pincemy G, Dobson FS, Jouventin P. Experiments on colour ornaments and mate choice in king penguins. Anim Behav. 2009; 78: 1247–1253. doi: 10.1016/j.anbehav.2009.07.041

[10] Nelson CM, Nolen TG. Courtship song, male agonistic encounters, and female mate choice in the house cricket, *Acheta domesticus* (Orthoptera: Gryllidae). J Insect Behav. 1997; 10: 557–570. doi: 10.1007/BF02765377

[11] Richardson C, Lengagne T. Multiple signals and male spacing affect female preference at cocktail parties in treefrogs. Pro R Soc B-Biol Sci. 2010; 277: 1247–1252. doi: 10.1098/rspb.2009.1836 20018785 10.1098/rspb.2009.1836 PMC2842810

[12] Ryan MJ, Rand AS. The sensory basis of sexual selection for complex calls in the tungara frog, *Physalaemus pustulosus* (Sexual Selection for Sensory Exploitation). Evolution 1990; 44: 305 doi: 10.1111/j.1558-5646.1990.tb05200.x 28564368 10.1111/j.1558-5646.1990.tb05200.x

[13] Passos C, Tassino B, Rosenthal GG, Reichard M. Reproductive behavior and sexual selection in annual fishes Annual fishes: life history strategy, diversity, and evolution. CRC Press, Boca Raton, FA, USA CRC Press (Boca Raton). 2015; 207–230. doi: 10.1201/b19016-16

[14] Rosenthal GG. Spatiotemporal dimensions of visual signals in animal communication. Annu Rev Ecol Evol S. 2007; 38: 155–178. doi: 10.1146/annurev.ecolsys.38.091206.095745

[15] Reale D, Reader SM, Sol D, Mcdougall PT, Dingemanse NJ. Integrating animal temperament within ecology and evolution. Biol Rev. 2007; 82: 291–318. doi: 10.1111/j.1469-185X.2007.00010.x 17437562 10.1111/j.1469-185X.2007.00010.x

[16] Bierbach D, Sommer-Trembo C, Hanisch J, Wolf M, Plath M. Personality affects mate choice: bolder males show stronger audience effects under high competition. Behav Ecol. 2015; 26: 1314–1325. doi: 10.1093/beheco/arv079

[17] Schuett W, Tregenza T, Dall SRX. Sexual selection and animal personality. Biol Rev. 2010; 85: 217–246. doi: 10.1111/j.1469-185X.2009.00101.x 10.1111/j.1469-185X.2009.00101.x 19922534

[18] Sommer-Trembo C, Bierbach D, Arias-Rodriguez L, Verel Y, Jourdan J, Zimmer C, et al Does personality affect premating isolation between locally-adapted populations? BMC Evol Biol. 2016; 16: 138 doi: 10.1186/s12862-016-0712-2 27338278 10.1186/s12862-016-0712-2 PMC4918032

[19] Godin JG, Dugatkin LA. Female mating preference for bold males in the guppy, *Poecilia reticulata*. P Natl Acad Sci USA. 1996; 93: 10262–10267. doi: 10.1073/pnas.93.19.10262 10.1073/pnas.93.19.10262 PMC38372 11607706

[20] Doutrelant C, McGregor PK. Eavesdropping and mate choice in female fighting fish. Behaviour 2000; 137: 1655–1668. doi: 10.1163/156853900502763

[21] Raine K, Petri TN, Anssi V, Jouni L. Females prefer bold males; an analysis of boldness, mate choice, andbacterial resistance in the field cricket *Gryllus integer*. Ecol Parasitol Immunol. 2012; 1: 1–6. doi: 10.4303/epi/235580

[22] Schuett W, Godin JJ, Dall SRX. Do female zebra finches, *Taeniopygia guttata*, choose their mates based on their 'personality'? Ethology. 2011; 117: 908–917. doi: 10.1111/j.1439-0310.2011.01945.x

[23] Kralj-Fišer S, Mostajo GAS, Preik O, Pekár S, Schneider JM. Assortative mating by aggressiveness type in orb weaving spiders. Behav Ecol. 2013; 24: 824–831. doi: 10.1093/beheco/art030

[24] Muraco JJ, Aspbury AS, Gabor CR. Does male behavioral type correlate with species recognition and stress? Behav Ecol. 2014; 25: 200–205. doi: 10.1093/beheco/art106

[25] Schuett W, Dall SR, Royle NJ. Pairs of zebra finches with similar ‘personalities’ make better parents. Anim Behav. 2011; 81: 609–618. doi: 10.1016/j.anbehav.2010.12.006

[26] Laubu C, Dechaume-Moncharmont FX, Motreuil S, Schweitzer C. Mismatched partners that achieve postpairing behavioral similarity improve their reproductive success. Sci Adv. 2016; 2: e1501013 doi: 10.1126/sciadv.1501013 26973869 10.1126/sciadv.1501013 PMC4783125

[27] Laubu C, Schweitzer C, Motreuil S, Louâpre P. Dechaume-Moncharmont FX. Mate choice based on behavioural type: do convict cichlids prefer similar partners? Anim Behav. 2017; 126: 281–291. doi: 10.1016/j.anbehav.2017.02.020

[28] Lindholm AK, Breden F. Sex chromosomes and sexual selection in poeciliid fishes. Am Nat. 2002; 160: S214–224. doi: 10.1086/342898 18707478 10.1086/342898

[29] Pollux BJA, Meredith RW, Springer MS, Garland T, Reznick DN. The evolution of the placenta drives a shift in sexual selection in livebearing fish. Nature. 2014; 513: 233–236. doi: 10.1038/nature13451 25043015 10.1038/nature13451

[30] Heinenkay JL, Morris KE, Ryan NA, Byerley SL, Venezia RE, Peterson MN, et al A trade-off between natural and sexual selection underlies diversification of a sexual signal. Behav Ecol. 2015; 26: 533–542. doi: 10.1093/beheco/aru228

[31] Plath M, Parzefall J, Korner KE, Schlupp I. Sexual selection in darkness? Female mating preferences in surface- and cave-dwelling Atlantic mollies, *Poecilia mexicana* (Poeciliidae, Teleostei). Behav Ecol Sociobiol. 2004; 55: 596–601. doi: 10.1007/s00265-003-0750-9

[32] Schlupp I, Plath M. Male mate choice and sperm allocation in a sexual/asexual mating complex of *Poecilia* (Poeciliidae, Teleostei). Biol Letters. 2005; 1: 169–171. doi: 10.1098/rsbl.2005.0306 17148157 10.1098/rsbl.2005.0306 PMC1626217

[33] Pyke GH. Plague minnow or mosquito fish? a review of the biology and impacts of introduced gambusia species. Annu Rev Ecol Evol S. 2008; 39: 171–191. doi: 10.1146/annurev.ecolsys.39.110707.173451

[34] Wilson RS. Temperature influences the coercive mating and swimming performance of male eastern mosquitofish. Anim Behav. 2005: 70: 1387–1394. doi: 10.1016/j.anbehav.2004.12.024

[35] Killen SS, Croft DP, Salin K, Darden SK. Male sexually coercive behaviour drives increased swimming efficiency in female guppies. Funct Ecol. 2016; 30: 576–583. doi: 10.1111/1365-2435.12527 27478292 10.1111/1365-2435.12527 PMC4949636

[36] Bisazza A, Vaccari G, Pilastro A. Female mate choice in a mating system dominated by male sexual coercion. Behav Ecol. 2001; 12: 59–64. doi: 10.1093/oxfordjournals.beheco.a000379

[37] Morris MR, Rioscardenas O, Darrah A. Male mating tactics in the northern mountain swordtail fish (*Xiphophorus nezahualcoyotl*): coaxing and coercing females to mate. Ethology. 2008; 114: 977–988. doi: 10.1111/j.1439-0310.2008.01541.x

[38] Tudor MS, Morris MR. Frequencies of alternative mating strategies influence female mate preference in the swordtail *Xiphophorus multilineatus*. Anim Behav. 2011; 82: 1313–1318. doi: 10.1016/j.anbehav.2011.09.014

[39] Evans JP, Pilastro A, Schlupp I. Ecology and evolution of poeciliid fishes. Chicago University Press, Chicago 2011; doi: 10.7208/chicago/9780226222769.001.0001

[40] Rahman MM, Turchini GM, Gasparini C, Norambuena F, Evans JP. The expression of pre- and postcopulatory sexually selected traits reflects levels of dietary stress in guppies. Plos One. 2014; 9: e105856 doi: 10.1371/journal.pone.0105856 25170940 10.1371/journal.pone.0105856 PMC4149491

[41] Gasparini C, Pilastro A, Evans JP. Male genital morphology and its influence on female mating preferences and paternity success in guppies. Plos One. 2011; 6(7): e22329 doi: 10.1371/journal.pone.0022329 21799825 10.1371/journal.pone.0022329 PMC3142123

[42] Harris S, Ramnarine IW, Smith HG, Pettersson LB. Picking personalities apart: estimating the influence of predation, sex and body size on boldness in the guppy *Poecilia reticulata*. Oikos. 2010; 119: 1711–1718. doi: 10.1111/j.1600-0706.2010.18028.x

[43] Cote J, Fogarty S, Weinersmith KL, Brodin T, Sih A. Personality traits and dispersal tendency in the invasive mosquitofish (*Gambusia affinis*). Pro R Soc B-Biol Sci. 2010; 277: 1571–1579. doi: 10.1098/rspb.2009.2128 20071380 10.1098/rspb.2009.2128 PMC2871838

[44] Brown C, Jones F, Braithwaite V. In situ examination of boldness–shyness traits in the tropical poeciliid, Brachyraphis episcopi. Anim Behav. 2005; 70: 1003–1009. doi: 10.1016/j.anbehav.2004.12.022

[45] Trompf L, Brown C. Personality affects learning and trade-offs between private and social information in guppies, *Poecilia reticulata*. Anim Behav. 2014; 88: 99–106. doi: 10.1016/j.anbehav.2013.11.022

[46] Lindstrom K, Ranta E. Social preferences by male guppies, *Poecilia reticulata*, based on shoal size and sex. Anim Behav. 1993; 46: 1029–1031. doi: 10.1006/anbe.1993.1289

[47] Head ML, Vegatrejo R, Jacomb F, Jennions MD. Predictors of male insemination success in the mosquitofish (*Gambusia holbrooki*). Ecol Evol. 2015; 5: 4999–5006. doi: 10.1002/ece3.1775 26640677 10.1002/ece3.1775 PMC4662323

[48] Pyke G. A review of the biology of Gambusia *affinis* and *G*. *holbrooki*. Rev Fish Biol Fisheries. 2005; 15: 339–365. doi: 10.1007/s11160-006-6394-x

[49] Bisazza A, Marconato A, Marin G. Male mate preferences in the mosquitofish *Gambusia holbrooki*. Ethology. 2010; 83: 335–343. doi: 10.1111/j.1439-0310.1989.tb00541.x

[50] Gomes-Silva G, Liu K, Chen B-j, Plath M, Sommer-Trembo C. Does individual variation in male mate choice copying reflect differences in social responsiveness? Ann Biol Sci. 2017; 5: 25–36. doi: 10.2176/2348-1927.1000106

[51] Dosen LD, Montgomerie R. Female size influences mate preferences of male guppies. Ethology. 2004; 110: 245–255. doi: 10.1111/j.1439-0310.2004.00965.x

[52] Budaev SV. "Personality" in the guppy (*Poecilia reticulata*): a correlational study of exploratory behavior and social tendency. J Comp Psychol. 1997; 111: 399–411. doi: 10.1037//0735-7036.111.4.399

[53] Jones KA, Godin JJ. Are fast explorers slow reactors? Linking personality type and anti-predator behaviour. Pro R Soc B-Biol Sci. 2010; 277: 625–632. doi: 10.1098/rspb.2009.1607 19864291 10.1098/rspb.2009.1607 PMC2842688

[54] Wilson ADM, Godin JJ. Boldness and behavioral syndromes in the bluegill sunfish, *Lepomis macrochirus*. Behav Ecol. 2009; 20: 231–237. doi: 10.1093/beheco/arp018

[55] Mazue GPF, Dechaume-Moncharmont FX, Godin JJ. Boldness–exploration behavioral syndrome: interfamily variability and repeatability of personality traits in the young of the convict cichlid (*Amatitlania siquia*). Behav Ecol. 2015; 26: 900–908. doi: 10.1093/beheco/arv030

[56] Wisenden BD, Sailer CD, Radenic SJ, Sutrisno R. Maternal inheritance and exploratory-boldness behavioural syndrome in zebrafish. Behaviour. 2011; 148: 1443–1456. doi: 10.1163/156853911X616530

[57] Kurvers RHJM, Van Oers K, Nolet BA, Jonker RM, Van Wieren SE, Prins HHT, et al Personality predicts the use of social information. Ecology Letters 2010 13: 829–837. doi: 10.1111/j.1461-0248.2010.01473.x 20482585 10.1111/j.1461-0248.2010.01473.x

[58] Husak JF, Fox SF, Lovern MB, Van Den Bussche RA. Faster lizards sire more offspring: sexual selection on whole-animal performance. Evolution. 2006; 60: 2122–2130. doi: 10.1554/05-647.1 17133868

[59] Peterson CC, Husak JF. Locomotor performance and sexual selection: individual variation in sprint speed of collared lizards (*Crotaphytus collaris*). Copeia. 2006; 2006: 216–224. doi: 10.1643/0045-8511(2006)6[216:LPASSI]2.0.CO;2

[60] Trott AR, Donelson NC, Griffith LC, Ejima A. Song choice is modulated by female movement in Drosophila males. PLos One. 2012; 7: e46025 doi: 10.1371/journal.pone.0046025 23049926 10.1371/journal.pone.0046025 PMC3458092

[61] Farr L, Andrews RV. Rank-associated differences in metabolic rates and locomotor activity of dominant and subordinate *Peromyscus maniculatus*. Comp Biochem Phys A. 1978; 61: 401–406. doi: 10.1016/0300-9629(78)90123-8

[62] Gębczyński AK, Konarzewski M. Locomotor activity of mice divergently selected for basal metabolic rate: a test of hypotheses on the evolution of endothermy. J Evolution Biol. 2009; 22: 1212–1220. doi: 10.1111/j.1420-9101.2009.01734.x 19344384 10.1111/j.1420-9101.2009.01734.x

[63] Moretti EHU, Madelaire CB, Silva RJD, Mendonca MT, Gomes FR. The relationships between parasite intensity, locomotor performance, and body condition in adult toads (*Rhinella icterica*) from the wild. J Herpetol. 2014; 48: 277–283. doi: 10.1670/10-339

[64] Goater CP, Semlitsch RD, Bernasconi MV. Effects of body size and parasite infection on the locomotory performance of juvenile toads, *Bufo bufo*. Oikos. 1993; 66: 129–136. doi: 10.2307/3545205

[65] Barber I, Dingemanse NJ. Parasitism and the evolutionary ecology of animal personality. Philos T R Soc B. 2010; 365: 4077–4088. doi: 10.1098/rstb.2010.0182 21078659 10.1098/rstb.2010.0182 PMC2992744

[66] Webster JP. The effect of Toxoplasma gondii and other parasites on activity levels in wild and hybrid *Rattus norvegicus*. Parasitology. 1994; 109: 583–589. doi: 10.1017/S0031182000076460 7831094 10.1017/s0031182000076460

[67] Pasternak AF, Huntingford FA, Crompton DW. Changes in metabolism and behaviour of the freshwater copepod *Cyclops strenuus abyssorum* infected with *Diphyllobothrium* spp. Parasitology. 1995; 110: 395–399. doi: 10.1017/S0031182000064738 7753580 10.1017/s0031182000064738

[68] Kekäläinen J, Lai YT, Vainikka A, Sirkka I, Kortet R. Do brain parasites alter host personality?—experimental study in minnows. Behav Ecol Sociobiol. 2014; 68: 197–204. doi: 10.1007/s00265-013-1634-2

[69] Adriaenssens B, Johnsson JI. Natural selection, plasticity and the emergence of a behavioural syndrome in the wild. Ecol Lett. 2013; 16: 47–55. doi: 10.1111/ele.12011 23034098 10.1111/ele.12011

[70] Boon AK, Reale D, Boutin S. The interaction between personality, offspring fitness and food abundance in North American red squirrels. Ecol Lett. 2007; 10: 1094–1104. doi: 10.1111/j.1461-0248.2007.01106.x 17877738 10.1111/j.1461-0248.2007.01106.x

[71] Conrad JL, Weinersmith KL, Brodin T, Saltz J, Sih A. Behavioural syndromes in fishes: a review with implications for ecology and fisheries management. J Fish Biol. 2011; 78: 395–435. doi: 10.1111/j.1095-8649.2010.02874.x 21284626 10.1111/j.1095-8649.2010.02874.x

[72] Montiglio P, Wey TW, Chang AT, Fogarty S, Sih A. Multiple mating reveals complex patterns of assortative mating by personality and body size. J Anim Ecol. 2016; 85: 125–135. doi: 10.1111/1365-2656.12436 26332682 10.1111/1365-2656.12436

[73] Ariyomo TO, Watt PJ. Disassortative mating for boldness decreases reproductive success in the guppy. Behav Ecol. 2013; 24: 1320–1326. doi: 10.1093/beheco/art070

[74] Cote J, Fogarty S, Sih A. Individual sociability and choosiness between shoal types. Anim Behav. 2012; 83: 1469–1476. doi: 10.1016/j.anbehav.2012.03.019

[75] Sih A, Bell AM. Insights for behavioral ecology from behavioral syndromes. Adv Study Behav. 2008; 38: 227–281. doi: 10.1016/S0065-3454(08)00005-3 24991063 10.1016/S0065-3454(08)00005-3 PMC4075144

[76] Sih A, Giudice MD. Linking behavioural syndromes and cognition: a behavioural ecology perspective. Philos T R Soc B. 2012; 367: 2762–2772. doi: 10.1098/rstb.2012.0216 22927575 10.1098/rstb.2012.0216 PMC3427552

[77] Yan X, Zhenyu L, Gregg WP, Dianmo L. Invasive species in China—an overview. Biodivers Conserv. 2001; 10: 1317–1341. doi: 10.1023/A:1016695609745

[78] Deaton R. Factors influencing male mating behaviour in *Gambusia affinis* (Baird & Girard) with a coercive mating system. J Fish Biol. 2008; 72: 1607–1622. doi: 10.1111/j.1095-8649.2008.01827.x

[79] Lafleur DL, Lozano GA, Sclafani M. Female mate-choice copying in guppies, *Poecilia reticulata*: a re-evaluation. Anim Behav. 1997; 54: 579–586. doi: 10.1006/anbe.1996.0452 9299043 10.1006/anbe.1996.0452

[80] Pitcher TE, Neff BD, Rodd FH, Rowe L. Multiple mating and sequential mate choice in guppies: females trade up. P Roy Soc B-Biol Sci. 2003; 270: 1623–1629. doi: 10.1098/rspb.2002.2280 12908984 10.1098/rspb.2002.2280 PMC1691420

[81] Casner AM, Fackelman HC, Degtyareva O, Kight SL. Do female western mosquitofish, *Gambusia affinis*, prefer ornaments that males lack? Ethology. 2016; 122: 561–570. doi: 10.1111/eth.12507

[82] Hughes AL. Male size, mating success, and mating strategy in the mosquitofish *Gambusia affinis* (Poeciliidae). Behav Ecol Sociobiol. 1985; 17: 271–278. doi: 10.1007/BF00300146

[83] Hoysak DJ, Godin JJ. Repeatability of male mate choice in the mosquitofish, *Gambusia holbrooki*. Ethology. 2007; 113: 1007–1018. doi: 10.1111/j.1439-0310.2007.01413.x

[84] Polverino G, Cigliano C, Nakayama S, Mehner T. Emergence and development of personality over the ontogeny of fish in absence of environmental stress factors. Behav Ecol Sociobiol. 2016; 70: 2027–2037. doi: 10.1007/s00265-016-2206-z

[85] Franck D. Der Anteil des “Coolidge-Effektes” an der isolationsbedingten Zunahme sexueller Verhaltensweisen von *Poecilia sphenops*). Ethology. 1975; 38: 472–481. doi: 10.1111/j.1439-0310.1975.tb02020.x

[86] Plath M, Seggel U, Burmeister H, Heubel KU, Schlupp I. Choosy males from the underground: male mating preferences in surface- and cave-dwelling Atlantic mollies (*Poecilia mexicana*). Naturwissenschaften. 2006; 93: 103–109. doi: 10.1007/s00114-005-0072-z 16404589 10.1007/s00114-005-0072-z

[87] Scharnweber K, Plath M, Tobler M. Examination of boldness traits in sexual and asexual mollies (*Poecilia latipinna*, *P*. *formosa*). Acta Ethol. 2011; 14: 77–83. doi: 10.1007/s10211-011-0097-6

[88] Brown C, Braithwaite VA. Size matters: a test of boldness in eight populations of the poeciliid *Brachyraphis episcopi*. Anim Behav. 2004; 68: 1325–1329. doi: 10.1016/j.anbehav.2004.04.004

[89] Burns JG. The validity of three tests of temperament in guppies (*Poecilia reticulata*). J Comp Psychol. 2008; 122: 344–356. doi: 10.1037/0735-7036.122.4.344 19014258 10.1037/0735-7036.122.4.344

[90] Moretz JA, Martins EP, Robison BD. Behavioral syndromes and the evolution of correlated behavior in zebrafish. Behav Ecol. 2007; 18: 556–562. doi: 10.1093/beheco/arm011

[91] Buckingham JN, Wong BBM, Rosenthal GG. Shoaling decisions in female swordtails: how do fish gauge group size? Behaviour. 2007; 144: 1333–1346. doi: 10.1163/156853907782418196

[92] Plath M, Strecker U. Behavioral diversification in a young species flock of pupfish (*Cyprionodon* spp.): shoaling and aggressive behavior. Behav Ecol Sociobiol. 2008; 62: 1727–1737. doi: 10.1007/s00265-008-0601-9

[93] Bell AM, Hankison SJ, Laskowski KL. The repeatability of behaviour: a meta-analysis. Anim Behav. 2009; 77:771–783. doi: 10.1016/j.anbehav.2008.12.022 24707058 10.1016/j.anbehav.2008.12.022 PMC3972767

[94] Boulton K, Grimmer AJ, Rosenthal GG, Walling CA, Wilson AJ. How stable are personalities? A multivariate view of behavioural variation over long and short timescales in the sheepshead swordtail, *Xiphophorus birchmanni*. Behav Ecol Sociobiol. 2014; 68: 791–803. doi: 10.1007/s00265-014-1692-0

[95] Dochtermann NA. Behavioral syndromes: carryover effects, false discovery rates, and a priori hypotheses. Behav Ecol. 2010; 21: 437–439. doi: 10.1093/beheco/arq021

[96] Archard GA, Braithwaite VA. Increased exposure to predators increases both exploration and activity level in *Brachyrhaphis episcopi*. J Fish Biol. 2011; 78: 593–601. doi: 10.1111/j.1095-8649.2010.02880.x 21284637 10.1111/j.1095-8649.2010.02880.x

[97] Cote J, Fogarty S, Brodin T, Weinersmith KL, Sih A. Personality-dependent dispersal in the invasive mosquitofish: group composition matters. Pro R Soc B-Biol Sci. 2011; 278:1670–1678. doi: 10.1098/rspb.2010.1892 21068033 10.1098/rspb.2010.1892 PMC3081767

[98] Smith BR, Blumstein DT. Behavioral types as predictors of survival in Trinidadian guppies (*Poecilia reticulata*). Behav Ecol. 2010; 21: 916–926. doi: 10.1093/beheco/arq084

[99] Barber I, Wright HA. How strong are familiarity preferences in shoaling fish? Anim Behav. 2001; 61: 975–979. doi: 10.1006/anbe.2000.1665

[100] Baldauf SA, Kullmann H, Thunken T, Winter S, Bakker TCM. Computer animation as a tool to study preferences in the cichlid *Pelvicachromis taeniatus*. J Fish Biol. 2009; 75: 738–746. doi: 10.1111/j.1095-8649.2009.02347.x 20738572 10.1111/j.1095-8649.2009.02347.x

[101] Chouinard-Thuly L, Gierszewski S, Rosenthal GG, Reader SM, Rieucau G, Woo LK, et al Technical and conceptual considerations for using animated stimuli in studies of animal behavior. Curr Zool. 2016; 63: 5–19. doi: 10.1093/cz/zow104 29491958 10.1093/cz/zow104 PMC5804155

[102] Scherer U, Godin JGJ, Schuett W. Validation of 2D‐animated pictures as an investigative tool in the behavioural sciences: a case study with a West African cichlid fish, *Pelvicachromis pulcher*. Ethology. 2017; 123 doi: 10.1111/eth.12630

[103] Polverino G, Liao JC, Porfiri M. Mosquitofish (*Gambusia affinis*) preference and behavioral response to animated images of conspecifics altered in their color, aspect ratio, and swimming depth. Plos One. 2013; 8(1): e54315 doi: 10.1371/journal.pone.0054315 23342131 10.1371/journal.pone.0054315 PMC3546983

[104] Bierbach D, Penshorn M, Hamfler S, Herbert D, Appel J, Meyer P, et al Gradient evolution of body colouration in surface- and cave-dwelling *Poecilia mexicana* and the role of phenotype-assortative female mate choice. Biomed Res Int. 2013; 2013: 148348–148348. doi: 10.1155/2013/148348 24175282 10.1155/2013/148348 PMC3794506

[105] Kodricbrown A, Nicoletto PF. Female choice in the guppy (*Poecilia reticulata*): the interaction between male color and display. Behav Ecol Sociobiol. 2001; 50: 346–351. doi: 10.1007/s002650100374

[106] Scherer U, Kuhnhardt M, Schuett W. Different or alike? Female rainbow kribs choose males of similar consistency and dissimilar level of boldness. Anim Behav. 2017; 128: 117–124. doi: 10.1016/j.anbehav.2017.04.007

[107] Sato A, Karino K. Use of digitally modified videos to examine female mate preference for orange spot coloration of males in the guppy, *Poecilia reticulata*. Ichthyol Res. 2006; 53: 398–405. doi: 10.1007/s10228-006-0364-0

[108] Bischoff RJ, Gould JL, Rubenstein DI. Tail size and female choice in the guppy (*Poecilia reticulata*). Behav Ecol Sociobiol. 1985; 46 doi: 10.1007/BF00300143

[109] Kodric-Brown A. Female choice of multiple male criteria in guppies: interacting effects of dominance, coloration and courtship. Behav Ecol Sociobiol. 1993; 32 doi: 10.1007/BF00168825

[110] Walling CA, Royle NJ, Lindström J, Metcalfe NB. Do female association preferences predict the likelihood of reproduction? Behav Ecol Sociobiol. 2010; 65 doi: 10.1007/s00265-009-0869-4

[111] Brommer JE, Class B. Phenotypic correlations capture between-individual correlations underlying behavioral syndromes. Behav Ecol Sociobiol. 2017; 71: 50 doi: 10.1007/s00265-017-2278-4

[112] Bisazza A. Male competition, female mate choice and sexual size dimorphism in poeciliid fishes. Mar Freshw Behav Phy. 1993; 23: 257–286. doi: 10.1080/10236249309378869

[113] Wong RY, So P, Cummings ME. How female size and male displays influence mate preference in a swordtail. Anim Behav. 2011; 82 doi: 10.1016/j.anbehav.2011.06.024

[114] Marler CA, Ryan MJ. Origin and maintenance of a female mating preference. Evolution. 1997; 51: 1244–1248. doi: 10.1111/j.1558-5646.1997.tb03971.x 28565473 10.1111/j.1558-5646.1997.tb03971.x

[115] Chen T, Beekman M, Ward AJW. The role of female dominance hierarchies in the mating behaviour of mosquitofish. Biol Lett-Uk. 2011; 7: 343–345. doi: 10.1098/rsbl.2010.1020 21123247 10.1098/rsbl.2010.1020 PMC3097860

[116] Mcpeek MA. Mechanisms of sexual selection operating on body size in the mosquitofish (*Gambusia holbrooki*). Behav Ecol. 1992; 3: 1–12. doi: 10.1093/beheco/3.1.1

[117] Plath M. Male mating behavior and costs of sexual harassment for females in cavernicolous and extremophile populations of Atlantic mollies (*Poecilia mexicana*). Behaviour. 2008; 145: 73–98. doi: 10.1163/156853908782687241

[118] Schlupp I, McKnab R, Ryan MJ. Sexual harassment as a cost for molly females: bigger males cost less. Behaviour. 2001; 138 doi: 10.1163/15685390151074438

[119] Pilastro A, Benetton S, Bisazza A. Female aggregation and male competition reduce costs of sexual harassment in the mosquitofish *Gambusia holbrooki*. Anim Behav. 2003; 65: 1161–1167. doi: 10.1006/anbe.2003.2118

[120] Dadda M, Pilastro A, Bisazza A. Male sexual harassment and female schooling behaviour in the eastern mosquitofish. Anim Behav. 2005; 70: 463–471. doi: 10.1016/j.anbehav.2004.12.010

[121] Morris MR, Rios-Cardenas O, Brewer J. Variation in mating preference within a wild population influences the mating success of alternative mating strategies. Anim Behav. 2010; 79: 673–678. doi: 10.1016/j.anbehav.2009.12.018

[122] Rios-Cardenas O, Tudor MS, Morris MR. Female preference variation has implications for the maintenance of an alternative mating strategy in a swordtail fish. Anim Behav. 2007; 74: 633–640. doi: 10.1016/j.anbehav.2007.01.002

[123] Witte K, Noltemeier B. The role of information in mate-choice copying in female sailfin mollies (*Poecilia latipinna*). Behav Ecol Sociobiol. 2002; 52: 194–202. doi: 10.1007/s00265-002-0503-1

[124] Servedio MR, Sæther SA, Sætre GP. Reinforcement and learning. Evol Ecol. 2009; 23: 109–123. doi: 10.1007/s10682-007-9188-2

[125] Schlupp I, Marler C, Ryan MJ. Benefit to male sailfin mollies of mating with heterospecific females. Science. 1994; 263: 373 doi: 10.1126/science.8278809 8278809 10.1126/science.8278809

[126] Zimmer C, Gavalas AS, Kunkel B, Hanisch J, Martin S, Bischoff S, et al Mate choice copying in both sexes of the guppy: The role of sperm competition risk and sexual harassment Sexual selection: evolutionary perspectives, mating strategies and long-term effects on genetic variation. Nova Science Publishers, Hauppauge 2013; NY: 69–92.

[127] Plath M, Bierbach D. Sex and the public: social eavesdropping, sperm competition risk, and male mate choice. Commu Integr Bio. 2011; 4: 276–280. doi: 10.4161/cib.4.3.14916 21980557 10.4161/cib.4.3.14916 PMC3187885

[128] Sih A, Mathot KJ, Moiron M, Montiglio P, Wolf M, Dingemanse NJ. Animal personality and state–behaviour feedbacks: a review and guide for empiricists. Trends Ecol Evol. 2015; 30: 50–60. doi: 10.1016/j.tree.2014.11.004 25498413 10.1016/j.tree.2014.11.004

[129] Kurvers RHJM, Prins HHT, Van Wieren SE, Van Oers K, Nolet BA, Ydenberg RC. The effect of personality on social foraging: shy barnacle geese scrounge more. P Roy Soc B-Biol Sci. 2010; 277: 601–608. doi: 10.1098/rspb.2009.1474 19864281 10.1098/rspb.2009.1474 PMC2842682

[130] Kurvers RHJM, Adamczyk VMAP, Van Wieren SE, Prins HHT. The effect of boldness on decision-making in barnacle geese is group-size-dependent. P Roy Soc B-Biol Sci. 2011; 278: 2018–2024. doi: 10.1098/rspb.2010.2266 21123271 10.1098/rspb.2010.2266 PMC3107651

[131] Gabriel PO, Black JM. Behavioural syndromes, partner compatibility and reproductive performance in Steller’s jays. Ethology. 2012; 118: 76–86. doi: 10.1111/j.1439-0310.2011.01990.x

[132] Ihle M, Kempenaers B, Forstmeier W. Fitness benefits of mate choice for compatibility in a socially monogamous species. Plos Biol. 2015; 13 doi: 10.1371/journal.pbio.1002248 26366558 10.1371/journal.pbio.1002248 PMC4569426

[133] Jiang YX, Bolnick DI, Kirkpatrick M. Assortative mating in animals. Am Nat. 2013; 181: E125–E138. doi: 10.1086/670160 23669548 10.1086/670160

[134] Reznick DN, Butler MJ, Rodd H. Life-history evolution in guppies. VII. The comparative ecology of high- and low-predation environments. Am Nat. 2001; 157: 126–140. doi: 10.1086/318627 18707267 10.1086/318627

[135] Riesch R, Reznick DN, Plath M, Schlupp I. Sex-specific local life-history adaptation in surface- and cave-dwelling Atlantic mollies (*Poecilia mexicana*). Sci Rep-Uk. 2016; 6: 22968–22968. doi: 10.1038/srep22968 26960566 10.1038/srep22968 PMC4785371

[136] Biro PA, Post JR. Rapid depletion of genotypes with fast growth and bold personality traits from harvested fish populations. P Natl Acad Sci Usa. 2008; 105: 2919–2922. doi: 10.1073/pnas.0708159105 18299567 10.1073/pnas.0708159105 PMC2268560

[137] Bierbach D, Oster S, Jourdan J, Arias-Rodriguez L, Krause J, Wilson ADM, et al Social network analysis resolves temporal dynamics of male dominance relationships. Behav Ecol Sociobiol 68: 1–11. doi: 10.1007/s00265-014-1706-y

[138] Basolo AL. Variation between and within the sexes in body size preferences. Anim Behav. 2004; 68: 75–82. doi: 10.1016/j.anbehav.2003.07.019

[139] Franck D. Sex, color, and mate choice in guppies. Ethology. 2000;106: 381–382. doi: 10.1046/j.1439-0310.2000.00565.x

[140] Edward DA, Chapman T. The evolution and significance of male mate choice. Trends Ecol Evol. 2011;26: 647–654. doi: 10.1016/j.tree.2011.07.012 21890230 10.1016/j.tree.2011.07.012

