# Supplementary figures and images for: Personality differentially affects individual mate choice decisions in female and male Western mosquitofish (Gambusia affinis)
Source: PLoS One. 2018 May 15;13(5):e0197197. doi: 10.1371/journal.pone.0197197 (PMC5953439; doi:10.1371/journal.pone.0197197)

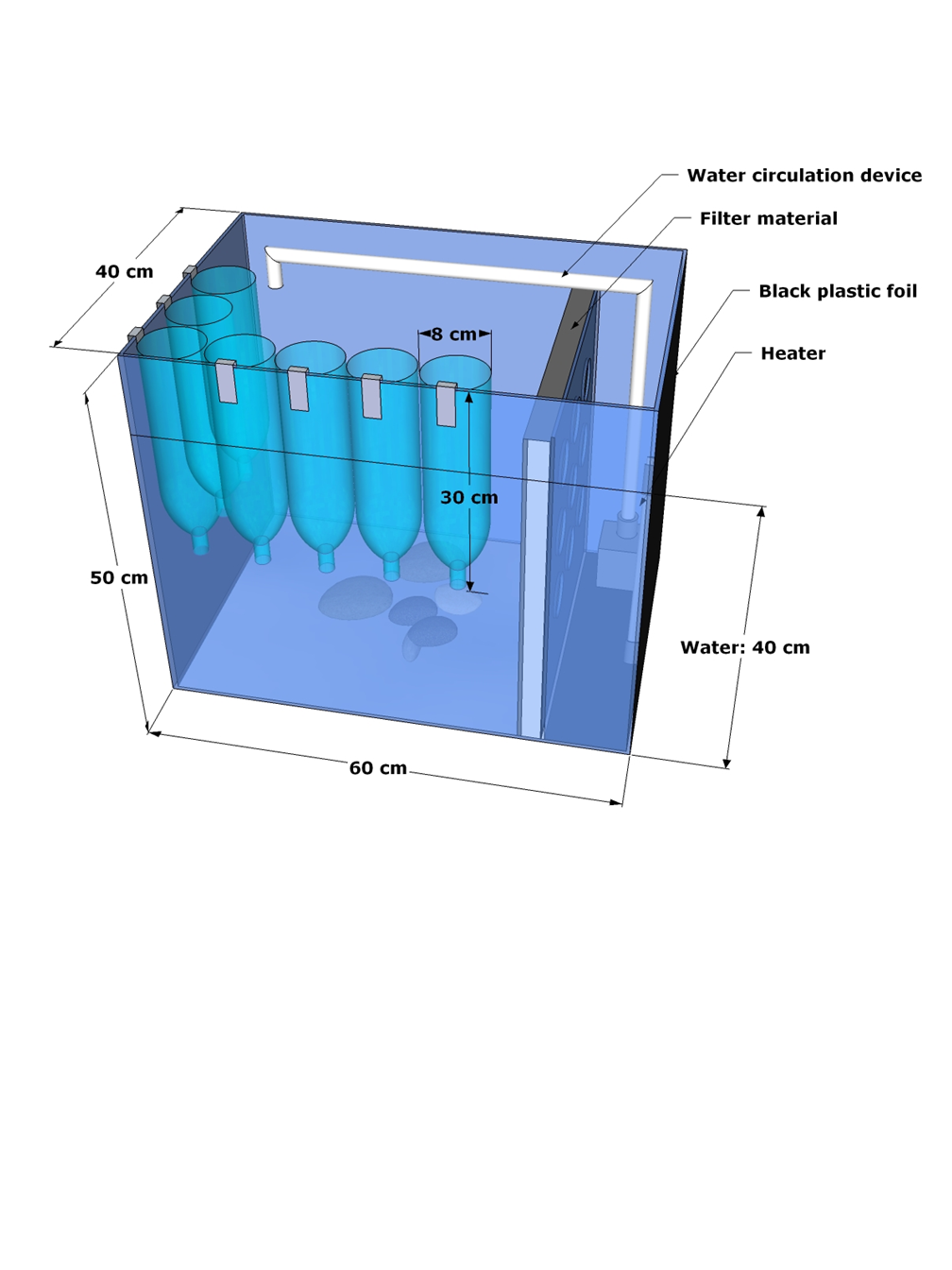

Supplement: S1 Fig — (TIF) [file pone.0197197.s001.tif]

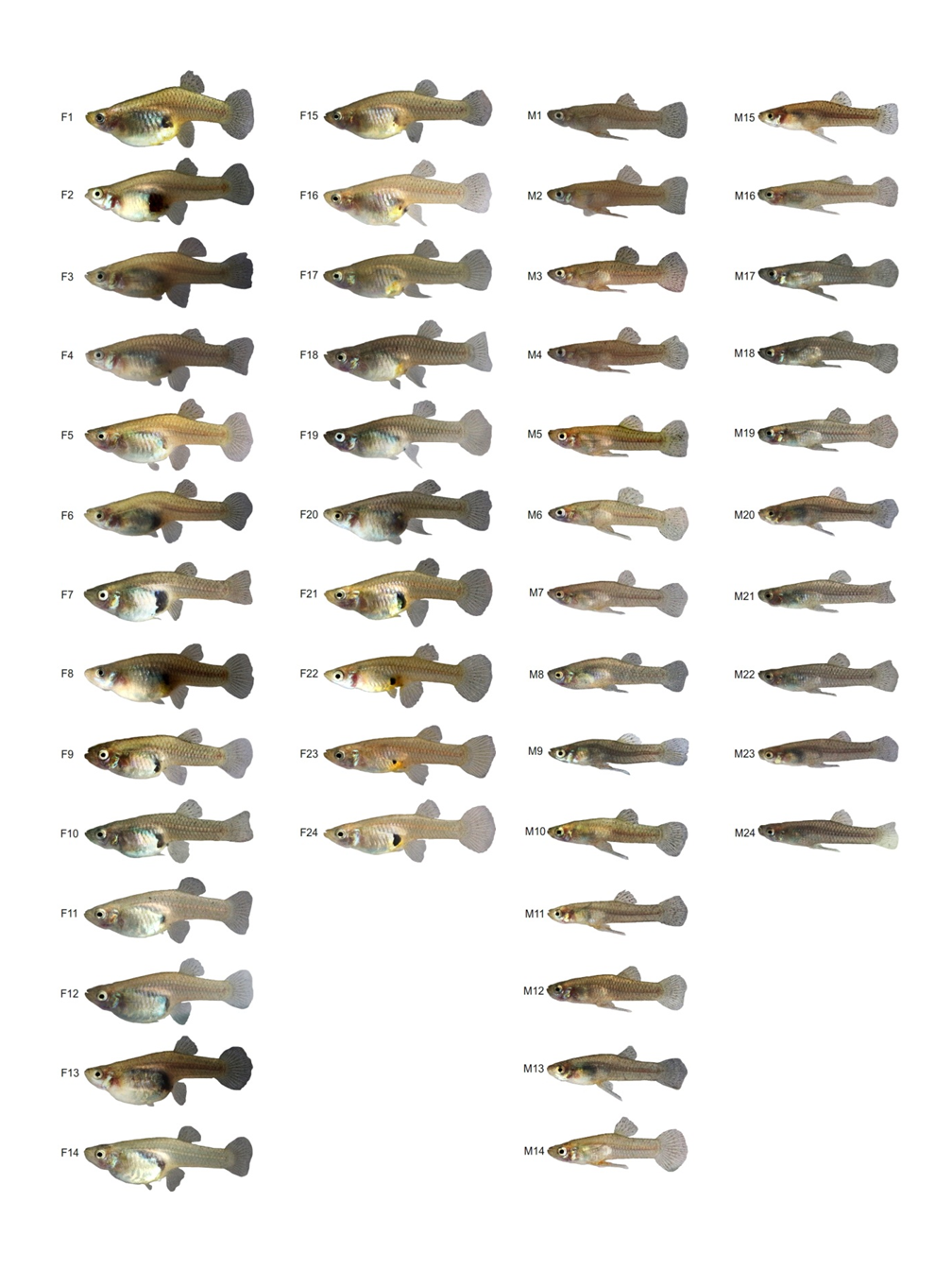

Supplement: S2 Fig — (TIF) [file pone.0197197.s002.tif]
