# Supplementary material for: Personality differentially affects individual mate choice decisions in female and male Western mosquitofish (Gambusia affinis)
Source: PLoS One. 2018 May 15;13(5):e0197197. doi: 10.1371/journal.pone.0197197 (PMC5953439; doi:10.1371/journal.pone.0197197)
Supplement: S1 Table — (DOCX) [file pone.0197197.s004.docx]

**Supporting information**

**Comparison of personality traits between sexes**

When comparing mean values from both personality assessments between sexes, we found females to show significantly higher shoaling tendencies than males, while sexes did not differ statistically in mean values of both other personality assessments (S1 Table). When testing for equality of variances of single personality trait between sexes, we found significantly higher variance in activity levels in females compared to males (S1 Table). By contrast, the variance in shoaling tendencies was higher in males than females, while sexes did not differ statistically in the case of our boldness assessment.

**S1 Table. Results from independent-samples *t*-tests comparing our three measures of personality between female and male *G. affinis.***

| Personality trait | Females | Males | Independent-  samples *t*-test | Levene’s test | 95% confidence interval | |
| --- | --- | --- | --- | --- | --- | --- |
| Boldness (emergence time) | 39.8 ± 6.6 s | 50.6 ± 9.0 s | *t*_82_ = –0.97  *P* = 0.34 | *F* = 2.53  *P* = 0.12 | -33.06 | 11.45 |
| Activity | 162.9 ± 11.3 sq | 150.7 ± 6.0 sq | *t*_62.38_ = 0.95  *P* = 0.35 | ***F* = 10.21**  ***P* = 0.002** | **-13.42** | **37.73** |
| Shoaling | 251.5 ± 7.8 s | 223.7 ± 11.4 s | ***t*_72.51_ = 2.02**  ***P* = 0.047** | ***F* = 6.67**  ***P* = 0.012** | **0.41** | **55.23** |

Boldness was estimated as time needed to emerge from shelter [s], activity as numbers of squares crossed [sq], and shoaling was measured as time spent in the vicinity of a stimulus shoal [s]. Levene’s tests were used to test for equality of variances. Significant differences are highlighted in bold.
