# Supplementary material for: Personality differentially affects individual mate choice decisions in female and male Western mosquitofish (Gambusia affinis)
Source: PLoS One. 2018 May 15;13(5):e0197197. doi: 10.1371/journal.pone.0197197 (PMC5953439; doi:10.1371/journal.pone.0197197)
Supplement: S2 Table — (DOCX) [file pone.0197197.s005.docx]

**Supporting information**

**Comparison of SOP-values between focal individuals larger or smaller and more or less active than the empirical mean**

If assortative mating occurs in our study species, large individuals should display significantly higher SOP-values for large-bodied mating partners than small ones. The same logic should apply when testing for focal individuals’ preference for more active mating partners: more active focal individuals should exhibit stronger preferences.

We divided focal individuals based in their SL and activity levels into cohorts that were smaller or larger than the empirical mean. In no case did a significant difference of SOPs between both groups become evident (*P* > 0.29, S2 Table) that would support assortative mating patterns.

**S2 Table. Results from independent-samples *t*-tests comparing SOP-values for large mating partners between large and small individuals and SOP-values for active mating partners between active and less active individuals in (*a*) female and (*b*) male *G. affinis*.**

| (*a*) Females | | | | |
| --- | --- | --- | --- | --- |
|  | Large | Small | Independent-samples *t*-test | Levene’s test |
| SOP for large mating partners | 0.14 ± 0.48 | 0.22 ± 0.36 | *t*_38_ = -0.60  *P* = 0.55 | *F* = 3.17  *P* = 0.083 |
|  | More active | Less active | Independent-samples *t*-test | Levene’s test |
| SOP for active mating partners | 0.033 ± 0.32 | -0.045 ± 0.43 | *t*_38_ = 0.62  *P* = 0.54 | *F* = 2.08  *P* = 0.16 |
| (*b*) Males | | | | |
|  | Large | Small | Independent-samples *t*-test | Levene’s test |
| SOP for large mating partners | -0.04 ± 0.38 | 0.079 ± 0.33 | *t*_40_ = -1.08  *P* = 0.29 | *F* = 0.78  *P* = 0.38 |
|  | More active | Less active | Independent-samples *t*-test | Levene’s test |
| SOP for active mating partners | 0.035 ± 0.30 | 0.11 ± 0.36 | *t*_40_ = -0.71  *P* = 0.48 | *F* = 0.75  *P* = 0.39 |
